# MAZ Regulates the Proliferation of Skeletal Muscle Satellite Cells via SLPI/Wnt-β-Catenin Signaling in Pigs

**DOI:** 10.3390/vetsci13070709

**Published:** 2026-07-19

**Authors:** Mengke Song, Rongru Zhu, Qian Zhang, Meng Li, Ming Tian, Xinmiao He, Xiuqin Yang

**Affiliations:** 1 College of Animal Science and Technology, Northeast Agricultural University, Harbin 150030, China; songmengke1113@163.com (M.S.); zhurongruzi@163.com (R.Z.); s210501020@neau.edu.cn (Q.Z.); 18864023292@163.com (M.L.); 2 Institute of Animal Husbandry, Heilongjiang Academy of Agricultural Sciences, Harbin 150086, China; tianming@haas.cn

**Keywords:** muscle growth, *SLPI*, *MAZ*, promoter, SNP

## Abstract

Skeletal muscle, accounting for about 40% of the body’s mass, is an important trait for farm animal production. Skeletal muscle satellite cells (MuSCs) are an excellent material for studying the regulatory mechanisms underlying muscle growth and regeneration after birth. Here, we analyzed the role and underlying mechanisms of Myc-associated zinc finger protein (MAZ) and secretory leukocyte protease inhibitor (SLPI) in porcine MuSC (pMuSC) proliferation for the first time. It was revealed that both *MAZ* and *SLPI* advanced the proliferation of pMuSCs by promoting cell cycle progression, and that a *MAZ/SLPI* axis regulated pMuSC proliferation. It was further shown that this *MAZ/SLPI* axis regulated pMuSC proliferation through the Wnt/β-catenin pathway. The proliferative role obtained above was confirmed in mouse myoblast C2C12 cells. Additionally, a single-nucleotide polymorphism (SNP), −360C>T, which frequently occurred in the promoter of the SLPI gene from Min, Yorkshire, and Longmin black pigs, was found to significantly increase the gene’s expression in vitro and in vivo. The SNP −360C>T in *SLPI* is a potential molecular marker predicting the proliferative ability of pMuSCs. The data provide a basis for controlling muscle growth and regeneration, which will provide benefits for improving the meat production of pigs and controlling muscle-related diseases in humans.

## Simple Summary

The growth and development of skeletal muscle is essential for porcine production as it directly determines meat yield and lean meat percentage. Muscle satellite cells (MuSCs) are closely associated with skeletal muscle growth through myogenesis. In the present study, the effects and the underlying mechanisms of *MAZ* and *SLPI* on the proliferation of porcine MuSCs (pMuSCs) were analyzed with gain- and loss-of-function methods. It was found that both *MAZ* and *SLPI* could increase the proliferation of pMuSCs by promoting cell cycle progression. Then, *SLPI* was shown to function as a downstream gene of *MAZ* during the regulation of pMuSCs proliferation. Furthermore, it was revealed that *MAZ/SLPI* regulated pMuSCs proliferation through canonical Wnt signaling. Additionally, a point mutation, SNP −360C>T, that frequently occurred in the promoter of porcine SLPI was shown to influence gene expression, suggesting potential as a molecular marker for pMuSCs proliferation.

## 1. Introduction

Skeletal muscle, accounting for about 40% of the body’s mass [1], is essential for livestock production. Its growth and development directly determine meat yield and lean meat percentage, and thus economic benefits. Additionally, its disorder results in many diseases such as muscular dystrophy/hypertrophy, muscular atrophy, and myosarcoma in humans [2]. Therefore, skeletal muscle is critical for both animal production and human medicine. It is important to reveal the mechanisms behind the growth and development of skeletal muscle, which is the basis for improving the meat production of farm animals and for controlling muscle-related diseases in humans. Researchers have long been working hard to unveil the mystery, and some progress has been made.

It has been revealed that skeletal myogenesis is an orchestrated process controlled by a series of transcription factors such as paired box (Pax) 3 and 5 and myogenic regulatory factors [2,3]. However, the mechanisms underlying muscle growth and development remain far from clarified. Muscle satellite cells (MuSCs), the skeletal muscle-resident stem cells, are an excellent material for studying the regulatory mechanisms underlying muscle growth and regeneration after birth. Their proliferative ability is closely associated with muscle mass and injury repair. Some genetic factors have been demonstrated to be involved in the regulation of MuSCs’ proliferation, including transcription factors, protein-coding genes, and noncoding RNAs [4,5,6].

The transcription factor Myc-associated zinc finger protein (MAZ) is extensively expressed in various tissues [7,8]. The polypeptide contains six C_2_H_2_-type zinc fingers responsible for binding to GC-rich sites within the promoter of the target genes at the carboxyl terminus. There is a proline-rich region consisting of 22% proline in the first 150 amino acids at the amino-terminal part. The proline-rich region serves as a transcriptional activation domain [7,9,10]. *MAZ* has been shown to be involved in every transcriptional process, including initiation, elongation, and termination [7,11], and is thus closely associated with the expression of many genes, including those modulating cell proliferation. For example, *MAZ* can activate the mitogen-activated protein kinase kinase 2 gene by directly binding to its promoter [12]. Studies also found that *MAZ* could transactivate muscle-specific genes including muscle creatine kinase, alpha-Actin, Desmin, and alpha-Myosin heavy chain in skeletal myocytes. Additionally, *MAZ*’s transcripts and DNA-binding activity were upregulated during skeletal myocyte differentiation [13]. However, the specific role of *MAZ* in MuSC growth and development remains to be identified.

Secretory leukocyte protease inhibitor (*SLPI*), a ~12 kDa inhibitor of cationic serine protease, is an important regulator of cell proliferation. It has shown a positive role in the proliferation of cells, including CD34+ hematopoietic progenitor cells [14,15], osteoblasts [16], etc. However, no studies on the role of SLPI in the proliferation of MuSCs were found. Although most studies have shown that SLPI promotes cell proliferation, opposite effects were found in a recent study [17], demonstrating the multiplicity and complexity of the SLPI gene’s actions in cell proliferation.

Our previous studies have revealed that *MAZ* and *SLPI* are differentially expressed during skeletal muscle development in pigs, suggesting that a *MAZ/SLPI* regulatory axis exists that regulates muscle growth and development. In the present study, we constructed a *MAZ/SLPI/Wnt/β-catenin* axis regulating the proliferation of porcine MuSCs (pMuSCs) and confirmed the proliferative role of the axis in mouse myoblast C2C12 cells. The results obtained here provide data further clarifying the mechanisms underlying pMuSCs’ proliferation, which will contribute to improving the meat production of farm animals and controlling muscle-related diseases in humans.

## 2. Materials and Methods

### 2.1. Animals and Nucleic Acids

*Min pig*, an indigenous pig breed unique to Northeast China, was used in the present study. Animals at the age of 210 d old were obtained from the Heilongjiang Academy of Agricultural Sciences, Harbin, China. The animals were reared in the same environment and had access to their diets and water ad libitum. The individuals were not related by blood, and gender was not taken into account in the present study. The pigs were slaughtered using electric stunning methods, and muscle tissues were collected. After being snap-frozen in liquid nitrogen, the tissues were stored at −80 °C. Animals were treated strictly based on the Chinese national standard for pig slaughtering (GB/T 17236–2019) [18], as approved by the Laboratory Animal Welfare and Ethics Committee of Northeast Agricultural University. RNA was isolated with Trizol reagent (Invitrogen, Carlsbad, CA, USA).

### 2.2. Cell Culture and Transfection

pMuSCs were purchased from Ming Zhou Biotechnology Co. (MZ-8377, MINGZHOUBIO, Ningbo, Zhejiang, China). pMuSCs were cultured in Dulbecco’s Modified Eagle’s Medium/Nutrient Mixture F-12 (DMEM/F12) containing 10% fetal bovine serum (FBS; Gibco BRL, Rockville, MD, USA) and 1% penicillin–streptomycin. C2C12 (CRL-1772) and PK-15 (CCL-33) cells were cultured in DMEM/High Glucose (SEVEN, Beijing, China) containing 10% FBS and 1% penicillin–streptomycin. Transient transfection was performed with Lipofectamine 2000 reagent (Invitrogen, Carlsbad, CA, USA) according to the manufacturer’s instructions. DNA was isolated with the normal phenol–chloroform method.

### 2.3. Reverse Transcription and PCRs

Reverse transcription was carried out with the HiScript III 1st Strand cDNA Synthesis Kit (+gDNA wiper) (Vazyme, Nanjing, China) to obtain cDNA. Regular PCR was carried out with 2× Taq PCR Master Mix (Vazyme). Real-time quantitative PCR (qPCR) was performed with the ChamQ Universal SYBR qPCR Master Mix (Vazyme) based on the manufacturer’s instructions, in triplicate. The 2^−ΔΔCt^ method was used to analyze the relative expression level of each gene, with β-actin as a reference. All primers were designed with primer premier 5.0 and synthesized by Genesoul Technology (Harbin, China). The sequences of all the primers used here are given in Table S1.

### 2.4. Plasmids and siRNA Sequences

An improved plasmid pCAGGS with modified enzyme sites at a polylinker provided by Dr. Honglin Jia, affiliated with the Harbin Veterinary Research Institute, Chinese Academy of Agricultural Sciences, was used to construct an overexpression vector. The complete coding sequences (CDSs) were first amplified from cDNA obtained from the muscle tissue of *Min pigs*, and then, sequences encoding three continuous hemagglutinin tags were spliced into the CDS with overlapping-extension PCR. The fragments were ligated into the improved pCAGGS at the SamI site with a ClonExpress II One Step Cloning Kit (Vazyme). Wild-type (WT) reporter genes containing the *SLPI* promoter were obtained previously [19]. The promoter was mutated with overlapping-extension PCR and inserted into pGL3-basic as described previously [20] to construct mutant type (MT) reporter genes. siRNAs against *MAZ* or *SLPI* were synthesized by General Biol (Hefei, China). The sequences of the primers and siRNAs are listed in Table S1.

### 2.5. Cell Counting Kit 8 and 5-Ethynyl-2′-Deoxyuridine Incorporation Assay

Cell counting kit 8 (CCK-8) was used, and 5-ethynyl-2′-deoxyuridine (EdU) staining was performed as described previously [21]. Briefly, overexpressing plasmids or siRNAs were transfected into the cells. The cells were collected at 0, 24, 48, 72, 96, and 120 h post-transfection and subjected to a CCK-8 assay according to the manufacturer’s instructions. The optical density (OD) was measured with a Tecan Microplate Reader Infinite F50 (Tean GENios, Mannendorf, Switzerland) at 450 nm. For the EdU assay, the cells were stained with the BeyoClick™ EdU-555 kit (Beyotime, Shanghai, China) according to the manufacturer’s protocol at 24 h post-transfection, and then visualized with the Olympus inverted fluorescence microscope IX71 (Olympus, Tokyo, Japan) at 346 nm (excitation)/460 nm (emission).

### 2.6. Western Blotting

Western blotting was carried out as described previously [20]. Briefly, when they reached 50% confluence, the cells were transfected and cultured for 48 h. Total protein was isolated with RIPA buffer (Beyotime, Shanghai, China) containing protease inhibitor (Invitrogen) and quantified with an enhanced BCA protein assay kit (Beyotime, Shanghai, China). A total of 25–30 µg of protein was separated by SDS–polyacrylamide gel electrophoresis and incubated with anti-PCNA (Proliferating cell nuclear antigen; 1:5000 dilution; Proteintech, Rosemont, IL, USA), β-tubulin (1:20,000 dilution; Proteintech), β-actin (1:20,000 dilution; Proteintech), or -HA tag (1:5000 dilution; Abmart, Shanghai, China). β-tubulin and -actin were used as references. Proteins were visualized with the Odyssey^®^ DLx near-infrared imaging system (LI-COR Biosciences, Lincoln, NE, USA). The intensities of the bands were measured with ImageStudio software (v5.2.2), and the relative values were calculated.

### 2.7. Flow Cytometry Analysis

The cells were cultured in six-well plates until 60% confluence and transfected for 24 h. After washing with phosphate-buffered solution (PBS), the cells were digested with trypsin and resuspended. The cells were stained with a cell cycle staining Kit (MultiSciences, Hangzhou, China) according to the manufacturer’s protocol. An Agilent Novo Cyte Flow Cytometer (Palo Alto, CA, USA) was used to analyze the cell cycle.

### 2.8. Dual-Luciferase Reporter Gene Analysis

Each of the reporter genes was co-transfected into PK-15 with the Renilla luciferase reporter, pRL-TK. At 48 h post-transfection, the luciferase activities were measured with the dual-luciferase reporter gene assay kit (Beyotime). The relative activity was calculated as the ratio of firefly to Renilla luciferase activity.

### 2.9. Polymorphism Analysis

The promoter of *SLPI* (Gene ID: 396886) was amplified, and single-nucleotide polymorphisms (SNPs) were identified through the direct sequencing of PCR products. SnapGene (v6.0.2) was used to scan the sequencing diagram to profile the genotypes of individuals. The diagram was manually inspected to confirm the SNPs and genotypes identified.

### 2.10. Statistical Analysis

All experiments were performed three times independently, each with triplicates. GraphPad Prism (version 9.5.1; GraphPad, San Diego, CA, USA) was used to analyze and visualize the data. Differences between two groups were compared with an unpaired t-test, and those between multiple groups were compared with one-way ANOVA. *, *p* < 0.05; **, *p* < 0.01.

## 3. Results

### 3.1. MAZ Promotes Skeletal Muscle Satellite Cell Proliferation

**Figure 1:**
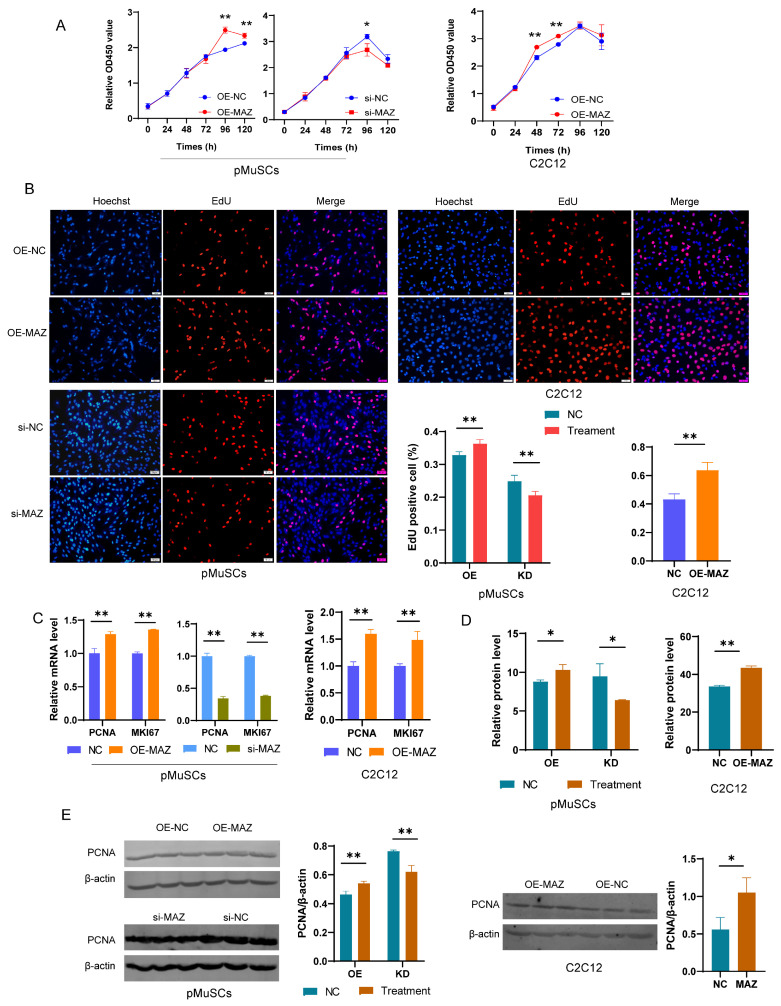
*MAZ* promotes muscle cell proliferation. (**A**,**B**) Effects of *MAZ* on muscle cell proliferation as revealed with CCK-8 (**A**) and EdU (**B**) assays. Red fluorescence indicates EdU-positive proliferating cells; blue fluorescence (Hoechst) labels all cell nuclei. Merge represents the overlay of EdU and Hoechst signals. (**C**) Effects of *MAZ* on expression of cell proliferation marker genes as revealed with qPCR. (**D**) Effects of *MAZ* on expression of *MKI67* as revealed with ELISA. (**E**) Effects of *MAZ* on expression of PCNA as revealed with Western blotting. OE, overexpression; KD, knockdown; si, RNAi; NC, negative control. *, *p* < 0.05; **, *p* < 0.01. The same as below. Scale bar: 50 μm.

Plasmids overexpressing *MAZ* and siRNA against MAZ functioned efficiently in pMuSCs, as revealed by Western blotting and qPCR assays (Figure S1). Compared to the negative control (NC), the overexpression of *MAZ* increased the cell viability of pMuSCs, while the knockdown of *MAZ* inhibited it, as revealed by CCK-8 assays (Figure 1A). EdU assays showed that overexpressing *MAZ* promoted DNA synthesis in pMuSCs, while knocking down *MAZ* weakened it (Figure 1B). Additionally, the expression of *MKI67* and *PCNA*, marker genes of cell proliferation, was increased by ectopic *MAZ* and decreased by knocking down *MAZ* in pMuSCs at the mRNA level (Figure 1C). The protein levels of MKI67 and PCNA changed similarly in response to *MAZ* treatment, as revealed by ELISA and Western blotting, respectively (Figure 1D,E). At the same time, C2C12 cells overexpressing *MAZ* were used to confirm the role of *MAZ* in cell viability and DNA synthesis, and consistent results were obtained (Figure 1).

### 3.2. MAZ Promotes Skeletal Muscle Satellite Cell Proliferation by Facilitating Cell Cycle Progression

**Figure 2:**
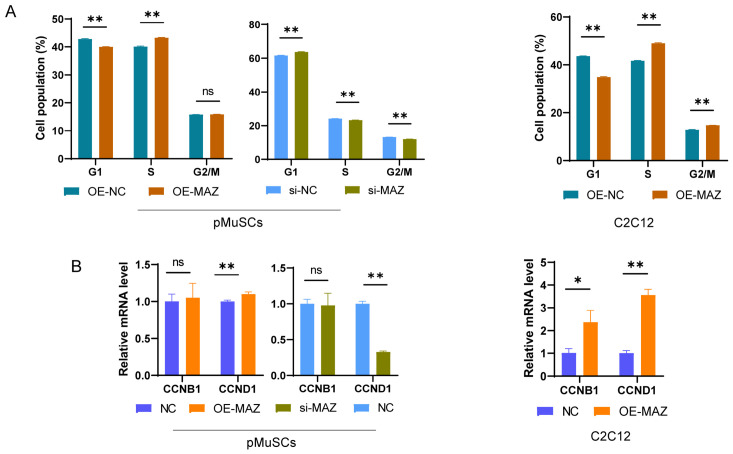
*MAZ* facilitates cell cycle progression. (**A**) Effects of *MAZ* on cell cycle progression. (**B**) Effects of *MAZ* on the expression of cell cycle regulators *CCND1* and *CCNB1*. OE, overexpression; si, RNAi; NC, negative control. *CCND1*, Cyclin D1; *CCNB1*, Cyclin B1; G1, Gap 1 phase; S, Synthesis phase; G2/M, Gap 2/Mitosis phase. *, *p* < 0.05; **, *p* < 0.01.

To analyze how *MAZ* affects the proliferation process of pMuSCs, the changes in cell cycle distribution were analyzed. Cells overexpressing *MAZ* showed reduced cell numbers in G0/G1-phase and increased numbers in S-phase compared to the NC groups in pMuSCs, while the knockdown of *MAZ* had opposite effects (Figure 2A). Similar results were obtained in C2C12 cells overexpressing *MAZ*, confirming the role of *MAZ* in cell proliferation (Figure 2A). To explore the mechanisms underlying the regulation of the cell cycle by *MAZ*, the expression changes in two representative cell cycle regulatory proteins—CCND1 and Cyclin B1 (CCNB1), which are G1/S- and G2/M-phase regulators, respectively—were measured in cells overexpressing or knocked down for *MAZ*. We found that *MAZ* promoted CCND1 levels’ stability in both pMuSCs and C2C12 cells. The changes in CCNB1 expression were not consistent across the two cell lines: *MAZ* had no effect on its expression in pMuSCs but enhanced it in C2C12 cells (Figure 2B), showing differences in the mechanisms behind the proliferative effects of MAZ between species. Nevertheless, the results indicated that *MAZ* promoted cell proliferation.

### 3.3. SLPI Functions as a Gene Downstream of MAZ to Regulate Skeletal Muscle Satellite Cell Proliferation

**Figure 3:**
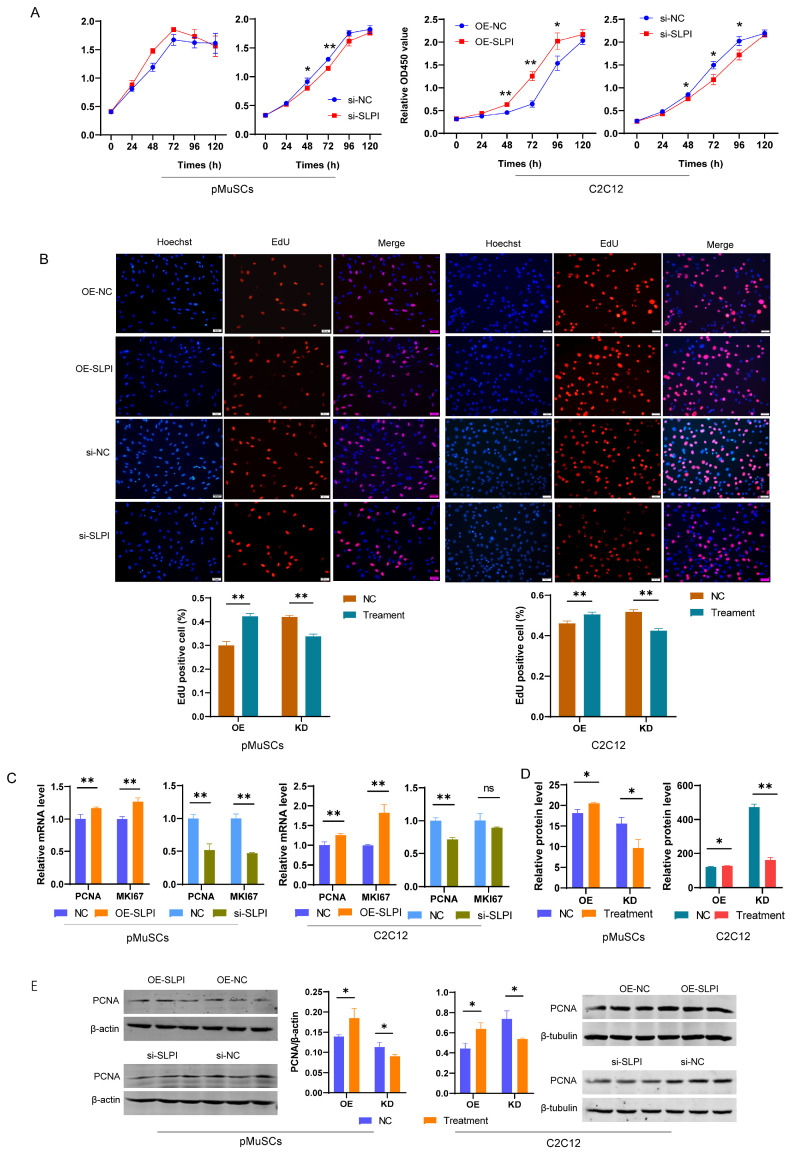
*SLPI* promotes muscle cell proliferation. (**A**,**B**) Effects of *SLPI* on muscle cell proliferation as revealed with CCK-8 (**A**) and EdU (**B**) assays. Red fluorescence indicates EdU-positive proliferating cells; blue fluorescence (Hoechst) labels all cell nuclei. Merge represents the overlay of EdU and Hoechst signals. (**C**) Effects of *SLPI* on expression of cell proliferation marker genes as revealed with qPCR. (**D**) Effects of *SLPI* on expression of *MKI67* as revealed with ELISA. (**E**) Effects of *SLPI* on expression of PCNA as revealed with Western blotting. OE, overexpression; si, RNAi; KD, knockdown; NC, negative control. *, *p* < 0.05; **, *p* < 0.01. Scale bar: 50 μm.

To analyze the role of *SLPI* in pMuSC proliferation, we first successfully constructed overexpression plasmids (Figure S2); the optimum siRNAs against *SLPI* in pMuSCs (si-173) or C2C12 (si-145) cells were also obtained (Figure S2). Studies in pMuSCs showed that ectopic expression of *SLPI* significantly increased cell viability and DNA synthesis, while knockdown of *SLPI* inhibited them (Figure 3A,B). Consistent results were obtained in C2C12 cells (Figure 3A,B). Furthermore, the expression changes in *PCNA* or *MKI67* in response to *SLPI* treatment further showed a positive role of *SLPI* in pMuSC proliferation because the overexpression of SLPI increased their expression at both the mRNA and protein levels, and knocking down *SLPI* decreased it in both cell lines (Figure 3C–E).

**Figure 4:**
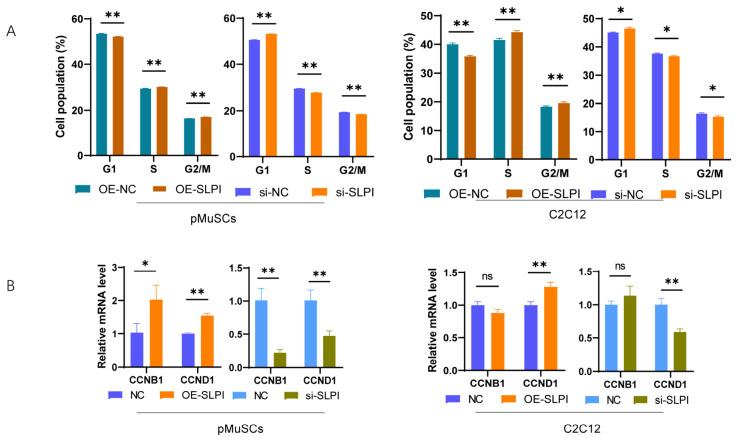
*SLPI* facilitates cell cycle progression. (**A**) Effects of *SLPI* on cell cycle progression. (**B**) Effects of *SLPI* on the expression of cell cycle regulators *CCND1* and *CCNB1*. OE, overexpression; si, RNAi; NC, negative control. *CCND1*, Cyclin D1; *CCNB1*, Cyclin B1; G1, Gap 1 phase; S, Synthesis phase; G2/M, Gap 2/Mitosis phase. *, *p* < 0.05; **, *p* < 0.01.

Cell cycle distribution analysis showed that overexpressing *SLPI* reduced the numbers of cells in G0/G1-phase and increased those in S- and G2/M-phase in both pMuSCs and C2C12 cells, and that opposite results were obtained when the expression of SLPI was knocked down (Figure 4A). The expression of *CCND1* and *CCNB1* was then analyzed in cells overexpressing or knocked down for *SLPI*, and the results showed that *SLPI* significantly enhanced the expression of CCND1 in both cells (Figure 4B).

**Figure 5:**
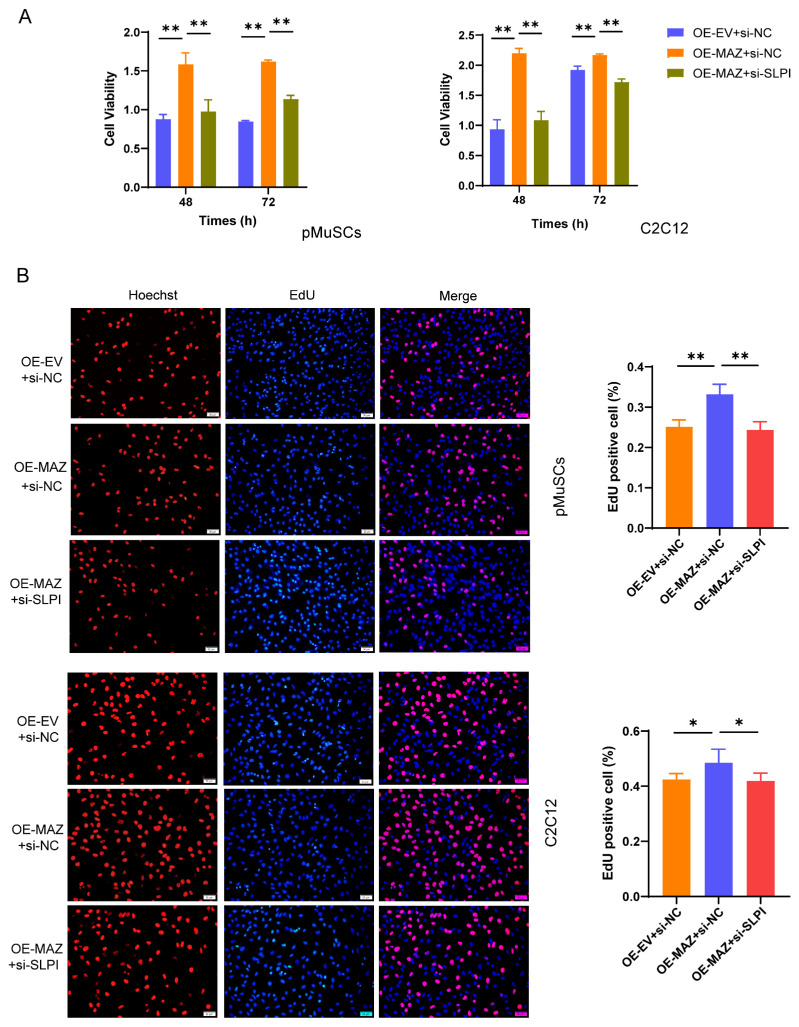
*SLPI* functions as a gene downstream of *MAZ* to promote cell proliferation. (**A**,**B**) Effects of co-transfection of *MAZ* and *SLPI* on muscle cell proliferation as revealed with CCK-8 (**A**) and EdU (**B**) assays. Red fluorescence indicates EdU-positive proliferating cells; blue fluorescence (Hoechst) labels all cell nuclei. Merge represents the overlay of EdU and Hoechst signals. OE, overexpression; si, RNAi; NC, negative control; EV, empty vector. *, *p* < 0.05; **, *p* < 0.01. Scale bar: 50 μm.

Rescue experiments showed that knocking down *SLPI* decreased the promoting effects of *MAZ* on the cell viability and DNA synthesis of pMuSCs according to CCK-8 and EdU assays, and the results were confirmed in C2C12 cells (Figure 5A,B), indicating that *MAZ* promotes MuSC proliferation via regulating *SLPI*.

### 3.4. MAZ/SLPI/Wnt/β-Catenin Axis Exists in Regulating Skeletal Muscle Satellite Cell Proliferation

**Figure 6:**
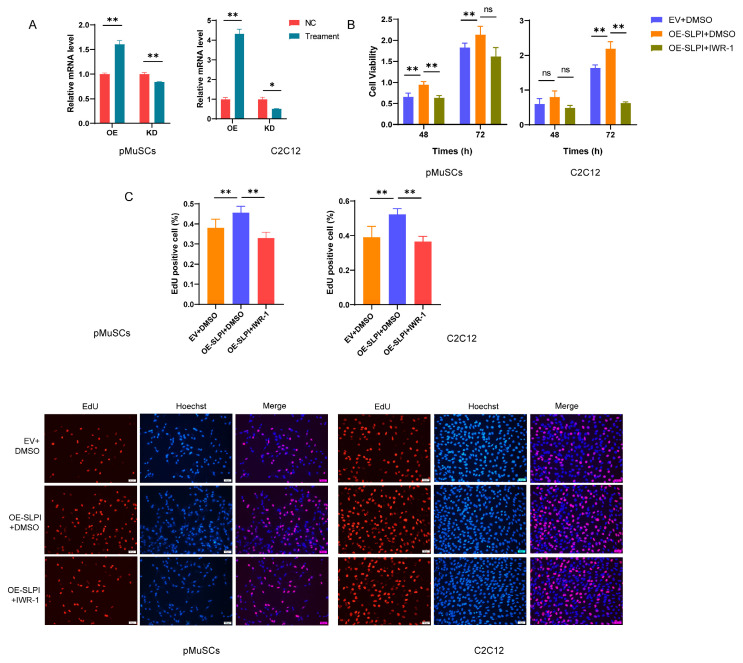
*MAZ/SLPI* axis regulates satellite cell proliferation through the Wnt-β-catenin pathway. (**A**) *SLPI* regulates the expression of β-catenin in muscle cells. (**B**,**C**) *SLPI* regulates muscle cell proliferation through the canonical Wnt pathway as revealed with CCK-8 (**B**) and EdU (**C**) assays. Red fluorescence indicates EdU-positive proliferating cells; blue fluorescence (Hoechst) labels all cell nuclei. Merge represents the overlay of EdU and Hoechst signals. In the panel: OE, overexpression; KD, knockdown; EV, empty vector; *, *p* < 0.05; **, *p* < 0.01. Scale bar: 50 μm.

The canonical Wnt/β-catenin pathway plays a pivotal role in regulating MuSC proliferation. We next investigated whether there was an association between the *MAZ/SLPI* axis and the Wnt/β-catenin pathway in regulating MuSC proliferation. As shown in Figure 6A, *SLPI* positively regulates the expression of β-catenin because overexpressing *SLPI* increased the expression of β-catenin, while knocking down *SLPI* decreased it in both pMuSCs and C2C12 cells. Further experiments revealed that IWR-1, an inhibitor of the canonical Wnt/β-catenin pathway, reversed the promoting effects of *SLPI* on the proliferation of pMuSCs and C2C12 cells, as revealed by CCK-8 and EdU assays (Figure 6B,C), confirming that Wnt/β-catenin is involved in the regulation of MuSC proliferation through the *MAZ/SLPI* axis.

### 3.5. Polymorphisms Affecting SLPI Expression

**Figure 7:**
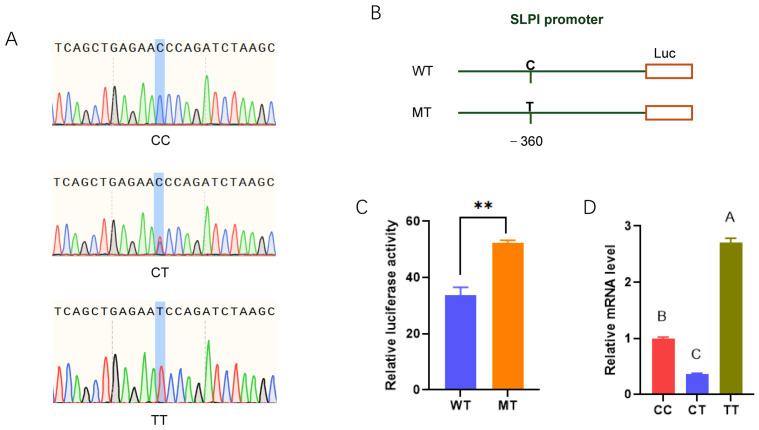
Single-nucleotide polymorphism (SNP) is associated with the expression of *SLPI*. (**A**) Sequencing diagram of three genotypes at the SNP −360C>T site. Blue corresponds to C, green to A, red to T, and black to G. (**B**) Schematic structure of reporter genes. (**C**) Effects of SNP −360C>T on the expression of reporter genes. (**D**) Effects of SNP −360C>T on endogenous expression of *SLPI*. **, *p* < 0.01. Bars with different letters indicate significant differences (*p* < 0.01).

SNP −360C>T was detected with high frequency in the promoter of *SLPI* by the direct sequencing of PCR products (Figure 7A). Genotyping analysis showed that the SNP frequently occurred in all three breeds/lines detected, including Min, a Chinese indigenous pig breed, and Yorkshire, *Yorkshire pig* and *LMB pigs*, Landrace × *Min pig* crossbred pigs. Allele T was dominant in *Min* and *Yorkshire pigs*, while in *LMB pigs*, allele C was dominant. Compared to *Yorkshire pigs*, allele C occurred more frequently in *Min pigs* (Table 1).

To reveal the role of SNP −360C>T in the expression of the *SLPI* gene, MT reporters containing the nucleotide T at position −360 were obtained based on WT reporters (Figure 7B). A C-to-T mutation significantly increased the promoter activity (Figure 7C). To further reveal the SNP’s impact on the expression of the *SLPI* gene, individuals with different genotypes were screened to measure the expression of *SLPI* in vivo. It was shown that genotype TT had the highest mRNA level of *SLPI* among the three genotypes (Figure 7D), which is consistent with the stronger promoter activity of reporter genes containing nucleotide T at that site.

## 4. Discussion

MuSCs, located between the plasma membrane and the basal lamina of myofibers, are a population of postnatal stem cells essential for skeletal muscle growth and regeneration through myogenesis [22,23,24,25], and they are therefore important for farm animal production and human muscle health. It is vital to reveal the mechanisms underlying the proliferation and differentiation of MuSCs. In the present study, it was revealed that *MAZ* promoted the proliferation of pMuSCs by facilitating cell cycle progression, and that *SLPI* functioned as a gene downstream of *MAZ* to regulate pMuSC proliferation. It was further revealed that the *MAZ/SLPI* axis regulated cell proliferation via canonical Wnt signaling. Additionally, an SNP influencing the expression of *SLPI* was identified in the promoter. The results enrich our knowledge of the regulatory mechanisms behind pMuSC proliferation and will contribute to controlling muscle growth and regeneration.

*MAZ* plays important roles during growth and development in mammals. As a transcriptional regulator, it has been demonstrated to be involved in various biological processes, including aerobic glycolysis [26], genitourinary development [27], ocular development [28], etc. Additionally, *MAZ* is involved in chromatin domain insulation and genome organization [29,30,31]. In the present study, it was first revealed that *MAZ* upregulated the proliferation of pMuSCs. Further analysis showed that *MAZ* could reduce cell numbers in G0/G1-phase, and thereafter facilitate cell cycle progression. The results extend the effects of *MAZ* to MuSCs, highlighting a role of *MAZ* in muscle growth and development.

A number of genes have been found to mediate the regulatory role of *MAZ* in cell proliferation, such as *LOC547*, *BRAF*, and *KRAS* [32,33]. In particular, *MAZ* could directly bind to the promoters of *BRAF* or *KRAS* to increase their transcription and expression levels during the proliferation of papillary thyroid carcinoma cells [33]. *MAZ* drives the tumor-specific expression of peroxisome proliferator-activated receptor gamma I, a regulator of cell cycle arrest [34,35,36]. However, the mechanism by which *MAZ* regulates pMuSCs’ proliferation remains to be further clarified. We found that *MAZ* might be a transcription factor for *SLPI* through the Assay for Transposase-Accessible Chromatin with High-Throughput Sequencing [19]. As a protease inhibitor, *SLPI* was first highlighted as a molecule that benefits the cell via its anti-proteolytic and immunomodulatory activities [37]. As research proceeds, increasing data have emerged to support this protein as a regulator of cell proliferation in various cells in humans [14,15,16,38]. However, no relationship between *SLPI* and the proliferation of MuSCs was found.

Thereafter, *SLPI* was selected as a candidate for mediating *MAZ*’s regulation of pMuSC proliferation. As expected, a promoting effect of SLPI on pMuSC proliferation was characterized, and *SLPI* was also shown to promote cell cycle progression. The similar effects of *MAZ* and *SLPI* indicate that there might be a relationship between them in regulating pMuSC proliferation. Through rescue experiments, it was confirmed that *SLPI* functions as a downstream gene regulating pMuSC proliferation because the promoting role of *MAZ* in cell proliferation was reversed when *SLPI* expression was inhibited. However, we did not make it clear whether *MAZ* regulated *SLPI* directly or indirectly here.

Additionally, it was found that both *MAZ* and *SLPI* could increase the expression of the *CCND1* gene. *CCND1*, an essential regulator of cell cycle progression, was tightly regulated by *MAZ/SLPI* during pMuSC proliferation, showing a positive correlation between *MAZ/SLPI* and *CCND1* expression. Studies have revealed that there was a high G/C content, characteristic of the *MAZ* binding motif, in the promoter of the human *CCND1* gene, especially in the region adjacent to the transcription initiation site, and that *MAZ* bound to the *CCND1* gene promoter by binding to the region [39]. There is also a G/C-rich region in the promoter of the porcine or mouse *CCND1* gene promoter; therefore, *MAZ* could be expected to directly transcriptionally regulate *CCND1* expression in the two species. Studies also indicated a link between *SLPI* and *CCND1* transactivation in that *SLPI* dose-dependently modulated the activity of the *CCND1* gene promoter in human cells [40]. Our results confirmed the regulatory effects of *SLPI* on *CCND1* in pigs and mice. However, it was not revealed whether *SLPI* functioned as a direct transcription factor to regulate *CCND1* gene expression. Further efforts should be made to help answer this question.

Wnt/β-catenin signaling is a key pathway regulating MuSC proliferation. Activation of the canonical Wnt signaling pathway robustly increases MuSC proliferation, and in activated MuSCs, the expression of β-catenin was strongly increased [41,42,43]. Additionally, β-catenin could bind the promoters of proliferative genes such as *CCND1* and c-myc via TCF/Lef transcription factors to activate their expression [42]. Thereafter, it was speculated that canonical Wnt signaling might be involved in *MAZ/SLPI*’s regulation of pMuSCs’ proliferation. As expected, β-catenin expression was significantly increased by ectopic *SLPI*, while it was decreased by silencing *SLPI*, indicating a link between the *SLPI* level and Wnt/β-catenin activation. Further rescue experiments showed that inhibiting Wnt/β-catenin signaling reversed the promoting effects of *SLPI* on satellite cell proliferation, validating that *SLPI* regulates pMuSC proliferation through the canonical Wnt signaling pathway.

Additionally, we identified a functional point mutation, SNP −360C>T, in the promoter of the *SLPI* gene, which frequently occurs among *Min*, *Yorkshire*, and *LMB pigs*. Through dual-luciferase reporter and qPCR assays, we found that a C-to-T mutation increased the promoter activity of *SLPI*, and pigs with the TT genotype had high levels of *SLPI* mRNA. Combining these findings with the result that *SLPI* promotes pMuSC proliferation, the SNP should be considered a molecular marker for predicting the proliferative ability of pMuSCs, contributing to controlling muscle growth and regeneration.

## 5. Conclusions

This study focused on revealing the role of *MAZ* and *SLPI* and exploring the relationship between them in the regulation of pMuSCs. Gain- and loss-of-function analysis showed that both *MAZ* and *SLPI* could advance pMuSC proliferation by promoting cell cycle progression, and a *MAZ/SLPI/Wnt-β-catenin* axis regulated pMuSC proliferation. Additionally, an SNP, −360C>T, was found to occur frequently in the promoter of the *SLPI* gene among pigs from *Min*, *Yorkshire*, and *LMB* breeds. It was further revealed that the SNP −360C>T could influence the expression of SLPI, indicating its potential as a molecular marker for predicting the proliferative ability of pMuSCs. These findings will contribute to controlling muscle growth and regeneration, which will benefit pig production and human muscle injury.

## Figures and Tables

**Table 1:** Distribution of SNP −360C>T in the promoter of SLPI among porcine populations.

Breed	Numbers	Genotype Frequency (Numbers)			Allele Frequency	
		CC	CT	TT	C	T
*Min*	137	0.25 (34)	0.29 (40)	0.46 (63)	0.39	0.61
*Yorkshire*	30	0.1 (3)	0.07 (2)	0.83 (25)	0.13	0.87
*LMB*	92	0.46 (42)	0.41 (38)	0.13 (12)	0.66	0.34

Notes: Min, *Min pig* (a Chinese indigenous pig breed); Yorkshire, *Yorkshire pig*; LMB, Landrace × *Min pig* crossbred pigs. *CC*, *CT* and *TT* represent three genotypes at the target locus. *C* and *T* refer to two alleles at the detected locus.

## Data Availability

The original contributions presented in this study are included in the article/Supplementary Materials. Further inquiries can be directed to the corresponding authors.

## Supplementary Materials

The following supporting information can be downloaded at: https://www.mdpi.com/article/10.3390/vetsci13070709/s1; Figure S1: Detection of MAZ overexpression and interference efficiency; Figure S2: Detection of SLPI overexpression and interference efficiency; Figure S3: Effects of MAZ on cell cycle progression; Figure S4: Effects of SLPI on cell cycle progression; Figure S5: Original Western blot images for detecting the overexpression efficiency of MAZ and SLPI; Figure S6: Original Western blot images showing the effects of MAZ on the protein expression levels of PCNA and β-actin in porcine muscle satellite cells (pMuSCs) and C2C12 cells; Figure S7: Original Western blot images showing the effects of SLPI on the expression levels of PCNA, β-actin and β-tubulin in porcine muscle satellite cells (pMuSCs) and C2C12 cells; Table S1: Oligonucleotide sequences used in this study.

## Abbreviations

The following abbreviations are used in this manuscript:

MuSC	Muscle satellite cell
MAZ	Myc-associated zinc finger protein
SLPI	Secretory leukocyte protease inhibitor
CCNB1	Cyclin B1
CCK-8	Cell counting kit 8
EdU	5-ethynyl-2′-deoxyuridine
PMuSCs	Porcine MuSCs
qPCR	Real-time quantitative PCR
CDS	Coding sequence
WT	Wild type
MT	Mutant type
SNPs	Single-nucleotide polymorphisms
Pax	Paired box
