# Supplementary material for: MAZ Regulates the Proliferation of Skeletal Muscle Satellite Cells via SLPI/Wnt-β-Catenin Signaling in Pigs
Source: Vet Sci. 2026 Jul 19;13(7):709. doi: 10.3390/vetsci13070709 (PMC13431503; doi:10.3390/vetsci13070709)
Supplement: Supplementary file 1 [file vetsci-13-00709-s001.zip › Supplementary Table S1 Oligonucleotide sequences.pdf]

Table S1 Primers used for amplification of coding sequences.

Names	Sequences (5’-3’)	Purpose
3HA-F	TGCATGGTACCCGGGTACCCGTACGACGTCCCG	Construction of plasmids overexpressing MAZ
3HA-MAZ-R	ACGGGGAACATGGCATAATCTGGAACATCGTAAG	
3HA-MAZ-F	AGATTATGCCATGTTCCCCGTGTTCCCTTG	
MAZ-pCAGGS-R	TCGAGCATGCCCGGGTCAGCAGGTGGGCTGTGG	
3HA-R	TCGAGCATGCCCGGGTCAGGCATAATCTGGAACATCGTAAG	Construction of plasmids overexpressing SLPI
SLPI-3HA-F	GTGAAAGCCTACCCGTACGACGTCCCG	
SLPI-pCAGGS-F	TGCATGGTACCCGGGATGGCACCTTGGGCCGT	
SLPI-3HA-R	GTACGGGTAGGCTTTCACAGGGGTGA	
siRNA-MAZ	CAGACAAGUGCACUCAACATT	Primers for siRNA interference detection
	UGUUGAGUGCACUUGUCUGTT	
siRNA-173	AAGAAAUGUUGCCGAGAUATT	
	UAUCUCGGCAACAUUUCUUTT	
siRNA-145	CCCAGUGCCUUAAGCUUGATT	
	UCAAGCUUAAGGCACUGGGTT	
siRNA-NC	UUCUCCGAACGUGUCACGUTT	
	ACGUGACACGUUCGGAGAATT	
SLPI-WT-F	TGTCCAGGGCTGAGGT	Construction of wild type gene
SLPI-WT-R	CCATTTTCAGCACCTTCCACGG	
－360C>T-F	GAGAATCCAGATCTAAGCAGC	For site-directed mutagenesis
－360C>T-R	TCTGGATTCTCAGCTGAGGC	
SLPI-sus-F	CAAGTGCACAAGTGACTGGC	Primers used for real-time quantitative PCR.
SLPI-sus-R	GGCCATAGACCACTGGACAC	
SLPI-mouse-F	TGAGAAGCCACAATGCCGTA	
SLPI-mouse-R	GGGAACAGGATTCACGCACT	
PCNA-sus-F	ATTTGGCCATGGGCGTGAAC	
PCNA-sus-R	CTAGTGCCAAGGTGTCTGCAT	
PCNA-mouse-F	GAAGTTTTCTGCAAGTGGAGAG	
PCNA-mouse-R	CAGGCTCATTCATCTCTATGGT	
MKI67-sus-F	ATTCCAGAAAGCACCAGGCT	
MKI67-sus-R	TCCACTGTCTTCTCAGGGGT	
MKI67-mouse-F	CCTGGTCTTAGTTCCGTTGA	
MKI67-mouse-R	GTTGGCGTTTCTCCTCTTTTC	
CCNB1-sus-F	AATCCCTTCTTGTGGTTA	
CCNB1-sus-R	CTTAGATGTGGCATACTTG	
CCNB1-mouse-F	AACTTCAGCCTGGGTCG	
CCNB1-mouse-R	CAGGGAGTCTTCACTGTAGGA	
CCND1-sus-F	TACACCGACAACTCCATCCG	
CCND1-sus-R	GAGGGCGGGTTGGAAATGAA	
CCND1-mouse-F	CGTATCTTACTTCAAGTGCGTG	

CCND1-mouse-R	ATGGTCTCCTTCATCTTAGAGG
β-catenin-sus-F	CTGTTCGCCTTCACTACGG
β-catenin-sus-R	GCTGGACAAAGGGCAAGA
β-catenin-mouse-F	GGGCAACCCTGAGGAAGAAGAT
β-catenin-mouse-R	CTTGCGTGAAGGACTGGGAAAA
