# Supplementary figures and images for: MAZ Regulates the Proliferation of Skeletal Muscle Satellite Cells via SLPI/Wnt-β-Catenin Signaling in Pigs
Source: Vet Sci. 2026 Jul 19;13(7):709. doi: 10.3390/vetsci13070709 (PMC13431503; doi:10.3390/vetsci13070709)

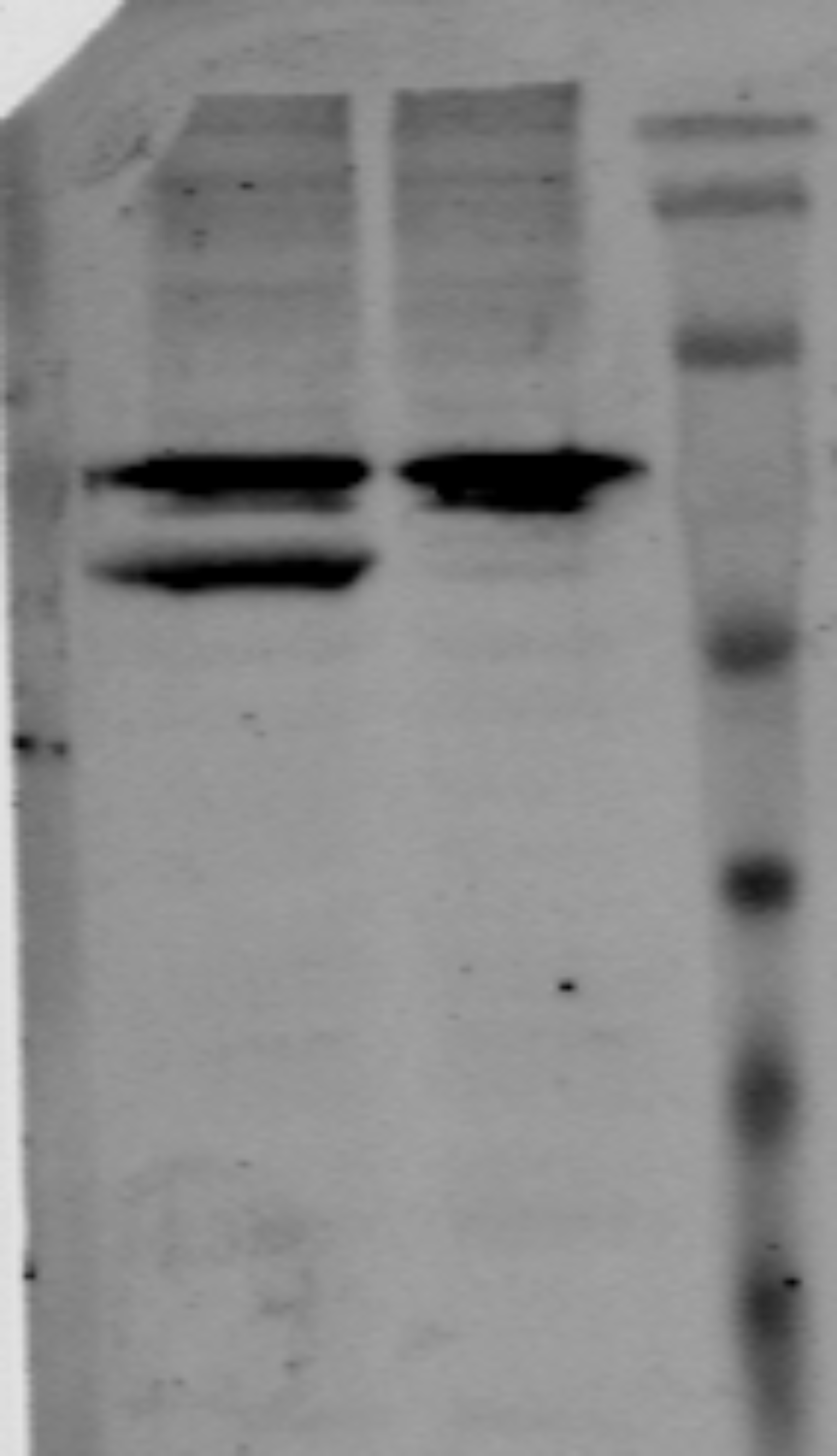

Supplement: Supplementary file 1 [file vetsci-13-00709-s001.zip › Supplementary Figure S1/Detection of MAZ overexpressed efficiency protein original image/oe-MAZ-HA-2.tiff]

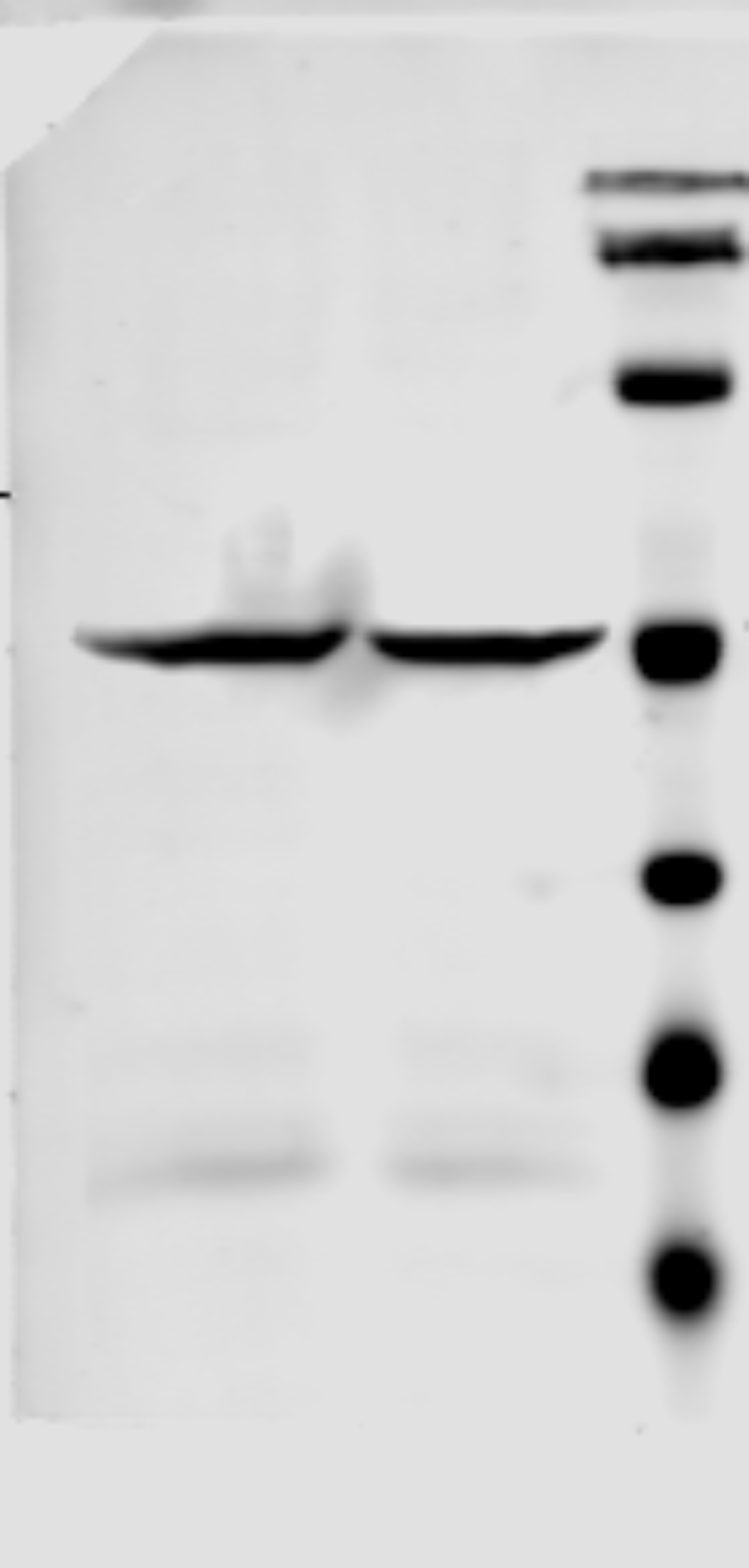

Supplement: Supplementary file 1 [file vetsci-13-00709-s001.zip › Supplementary Figure S1/Detection of MAZ overexpressed efficiency protein original image/oe-MAZ-tubulin-2.tiff]

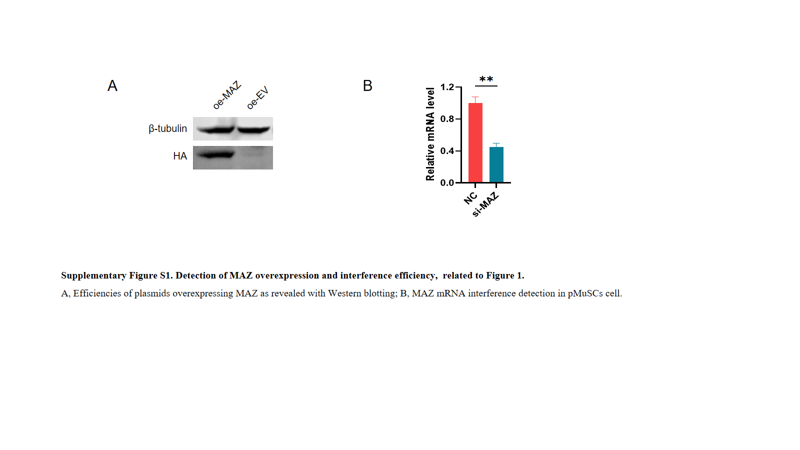

Supplement: Supplementary file 1 [file vetsci-13-00709-s001.zip › Supplementary Figure S1/Detection of MAZ overexpression and interference efficiency.png]

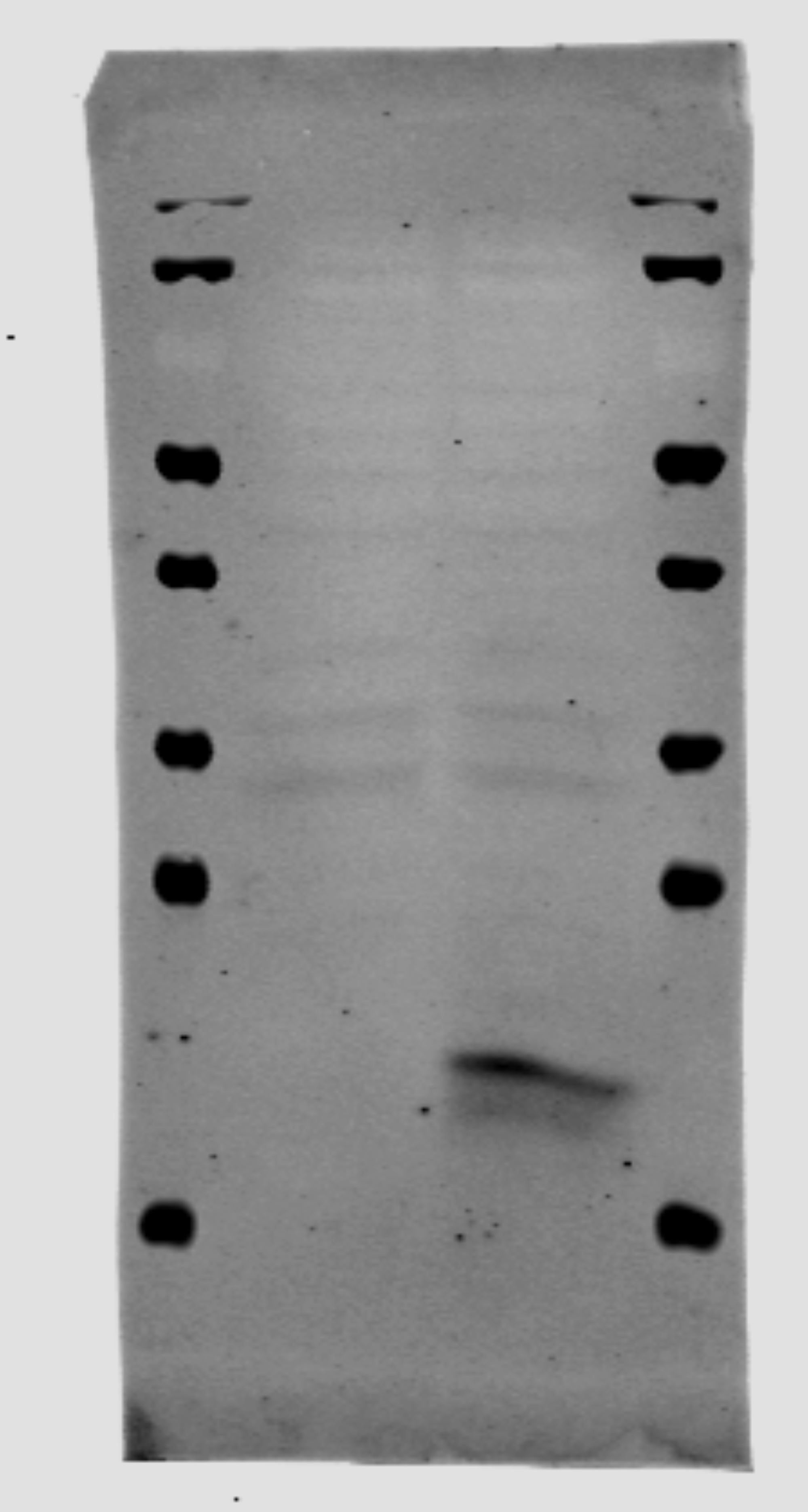

Supplement: Supplementary file 1 [file vetsci-13-00709-s001.zip › Supplementary Figure S2/Detection of SLP1 overexpressed efficiency protein original image/oe-SLPI-HA-1.tif]

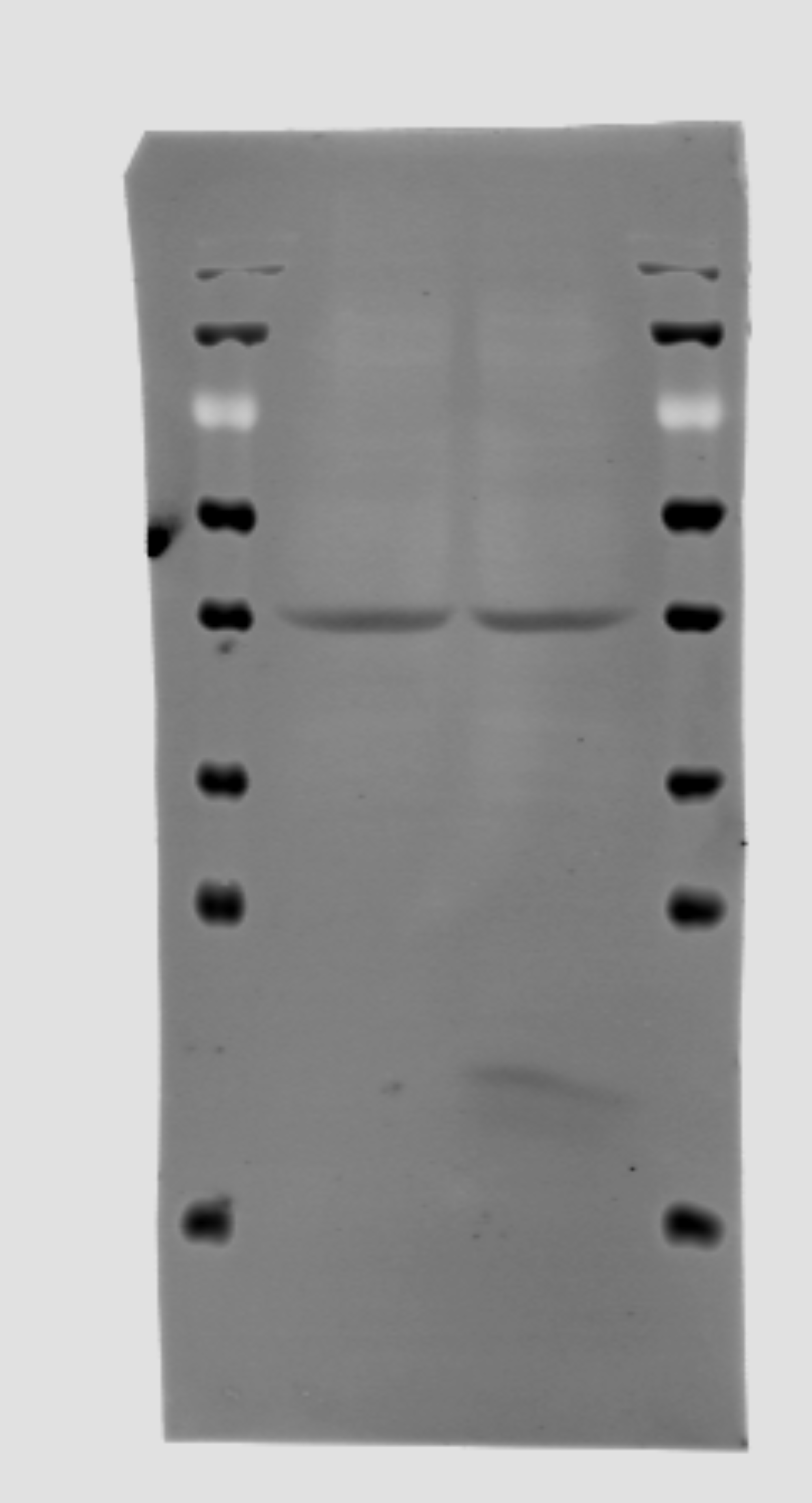

Supplement: Supplementary file 1 [file vetsci-13-00709-s001.zip › Supplementary Figure S2/Detection of SLP1 overexpressed efficiency protein original image/oe-SLPI-β-actin-1.tif]

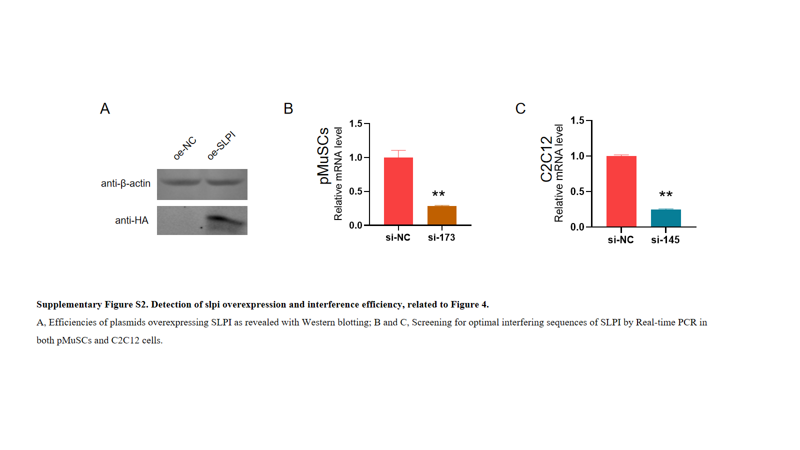

Supplement: Supplementary file 1 [file vetsci-13-00709-s001.zip › Supplementary Figure S2/Detection of slpi overexpression and interference efficiency.png]

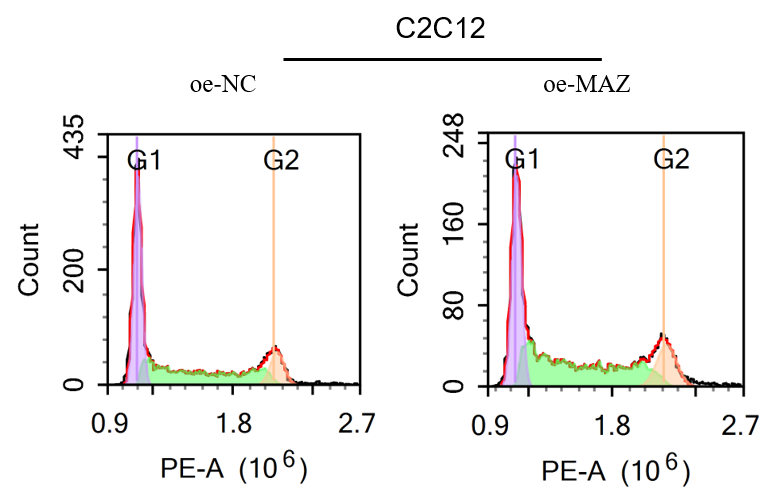

Supplement: Supplementary file 1 [file vetsci-13-00709-s001.zip › Supplementary Figure S3/Effects of MAZ on cell cycle progression/Effects of SLPI on cell cycle in C2C12 cells/oe-MAZ.tif]

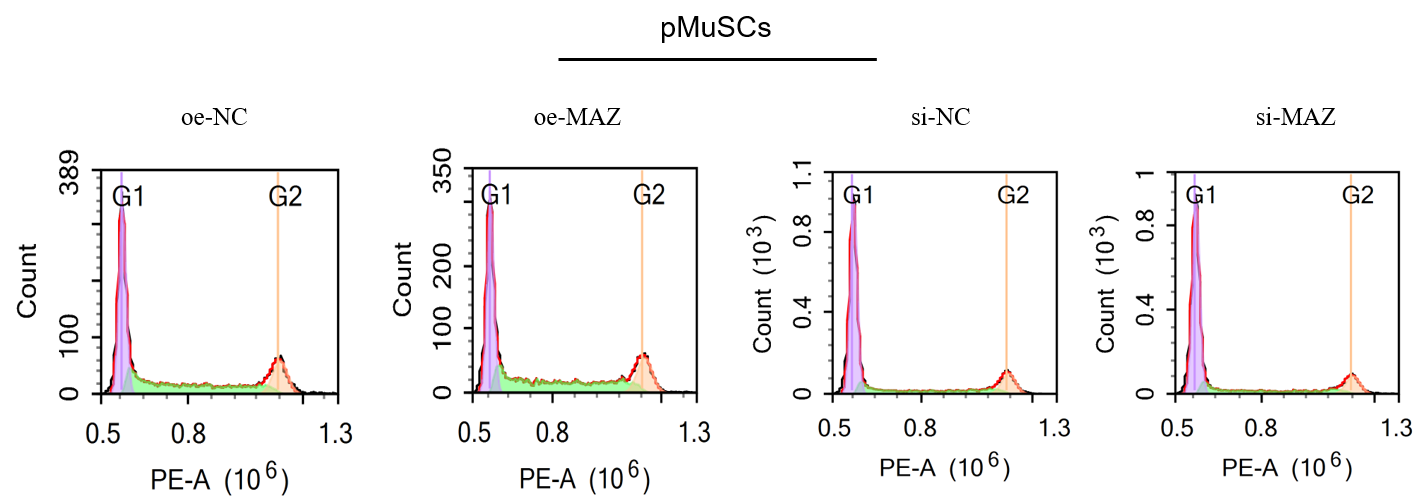

Supplement: Supplementary file 1 [file vetsci-13-00709-s001.zip › Supplementary Figure S3/Effects of MAZ on cell cycle progression/Effects of SLPI on cell cycle in pMuSCs cells/oe-MAZ.tif]

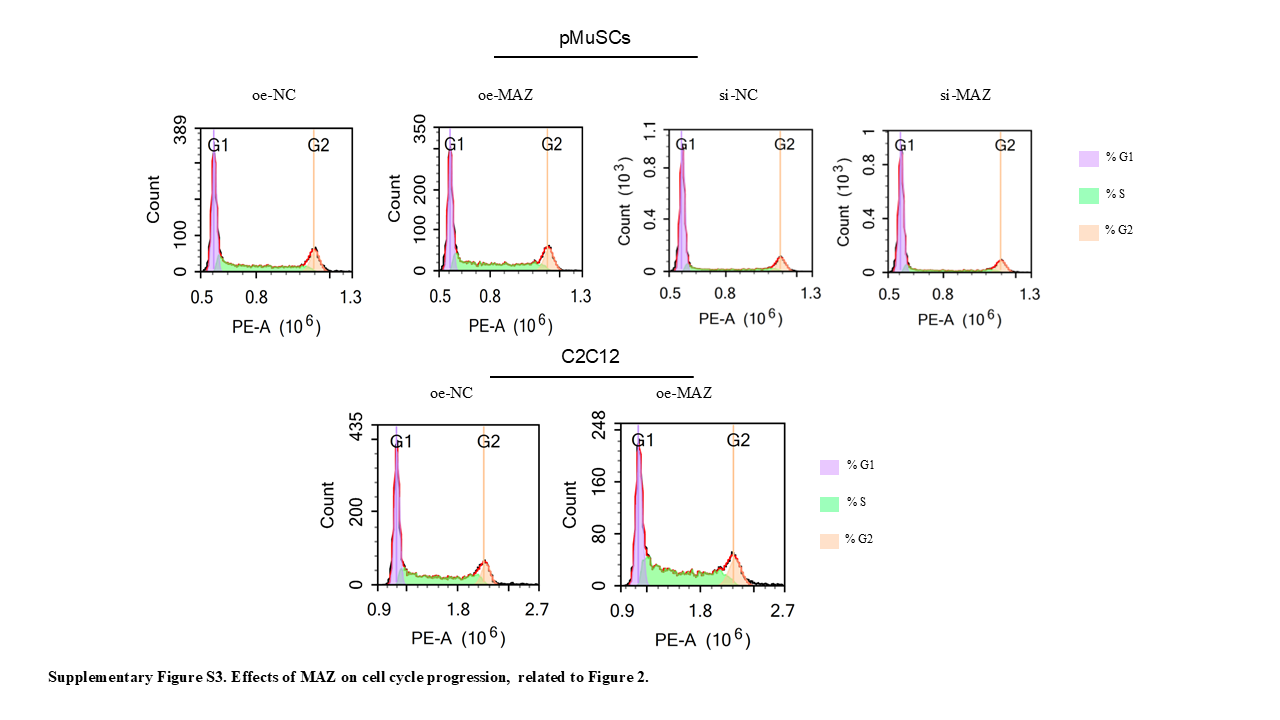

Supplement: Supplementary file 1 [file vetsci-13-00709-s001.zip › Supplementary Figure S3/Effects of MAZ on cell cycle progression/Figure S3 Effects of MAZ on cell cycle progression.tif]

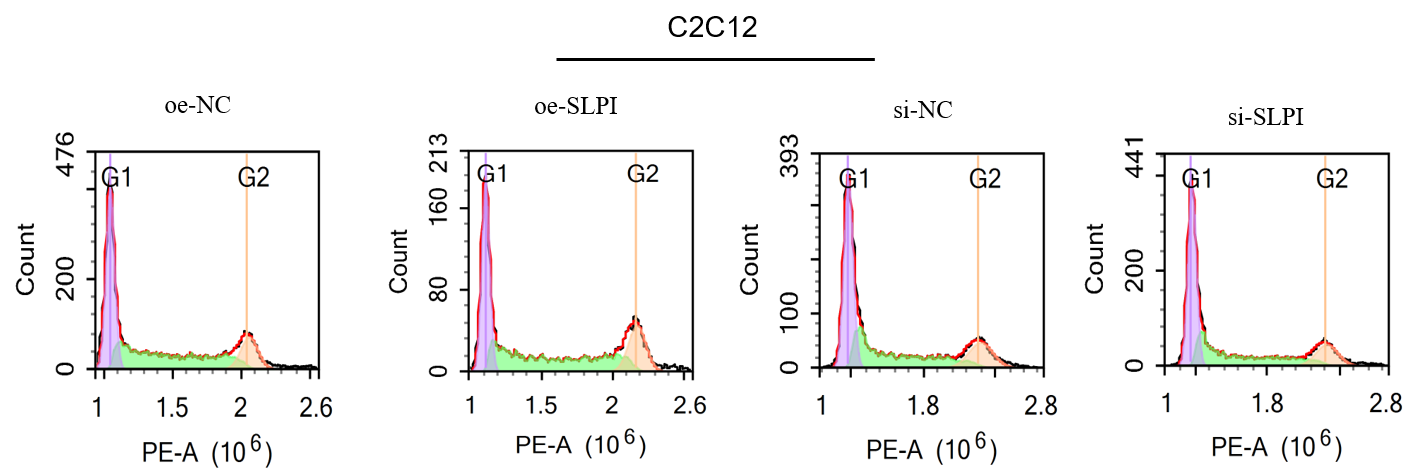

Supplement: Supplementary file 1 [file vetsci-13-00709-s001.zip › Supplementary Figure S4/Effects of SLPI on cell cycle in C2C12 cells/oe-NC.tif]

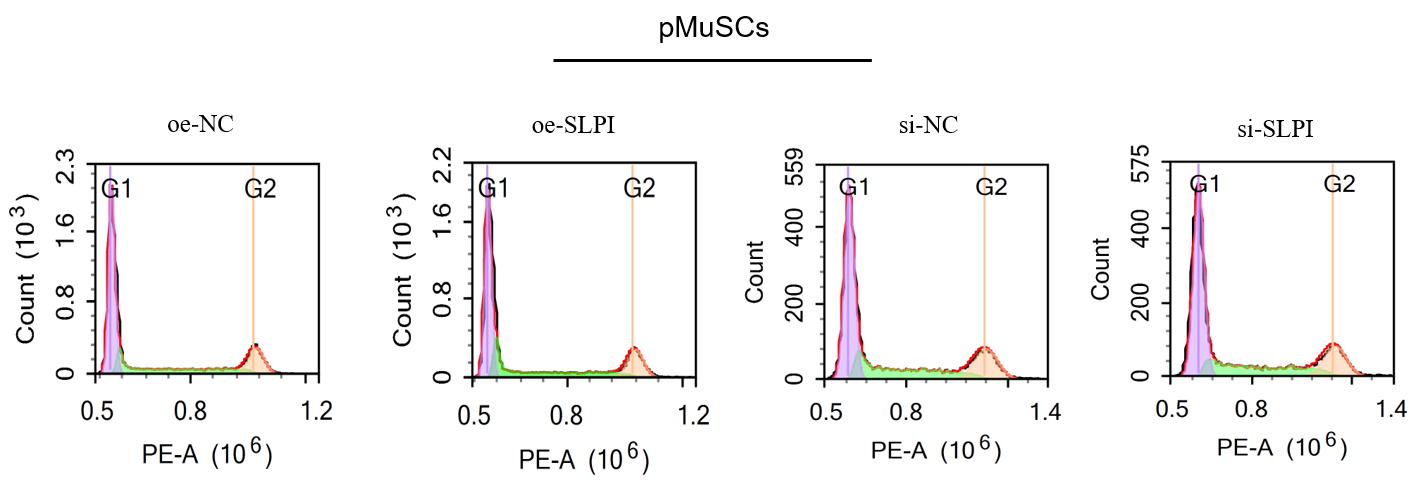

Supplement: Supplementary file 1 [file vetsci-13-00709-s001.zip › Supplementary Figure S4/Effects of SLPI on cell cycle in pMuSCs cells/oe-NC.tif]

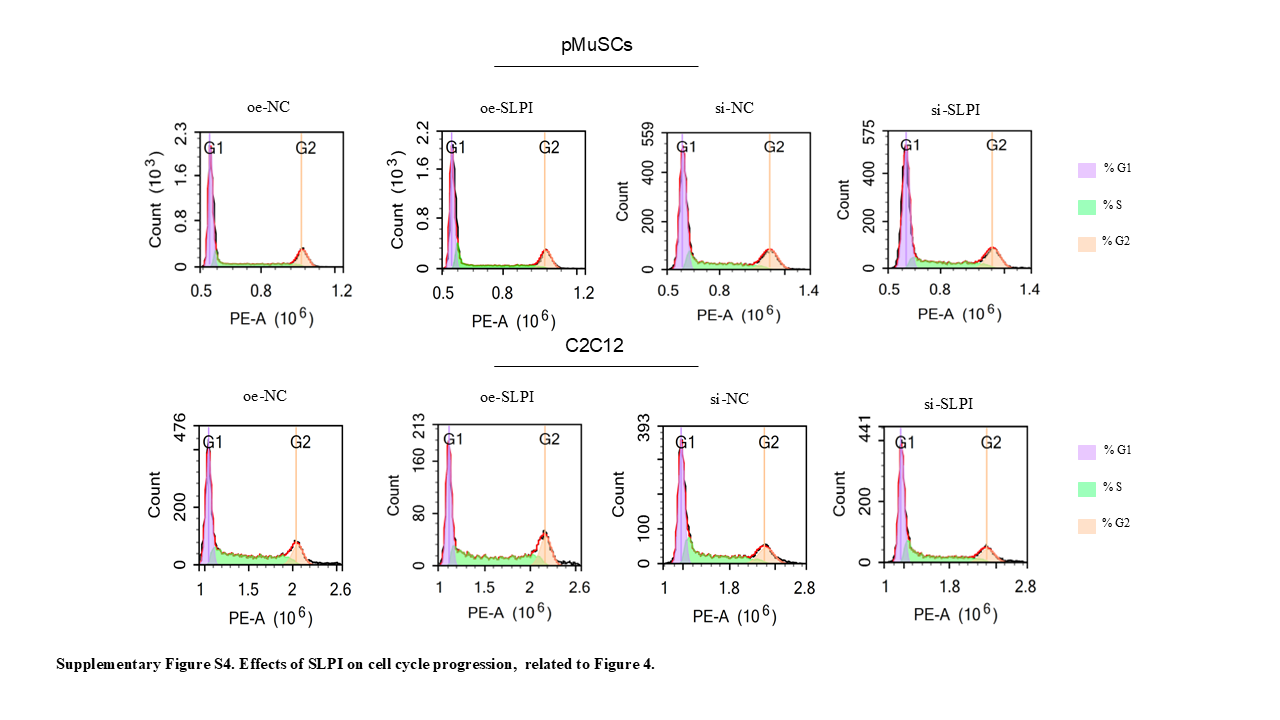

Supplement: Supplementary file 1 [file vetsci-13-00709-s001.zip › Supplementary Figure S4/Supplementary Figure S4.Effects of SLPI on cell cycle progression, related to Figure 4..tif]

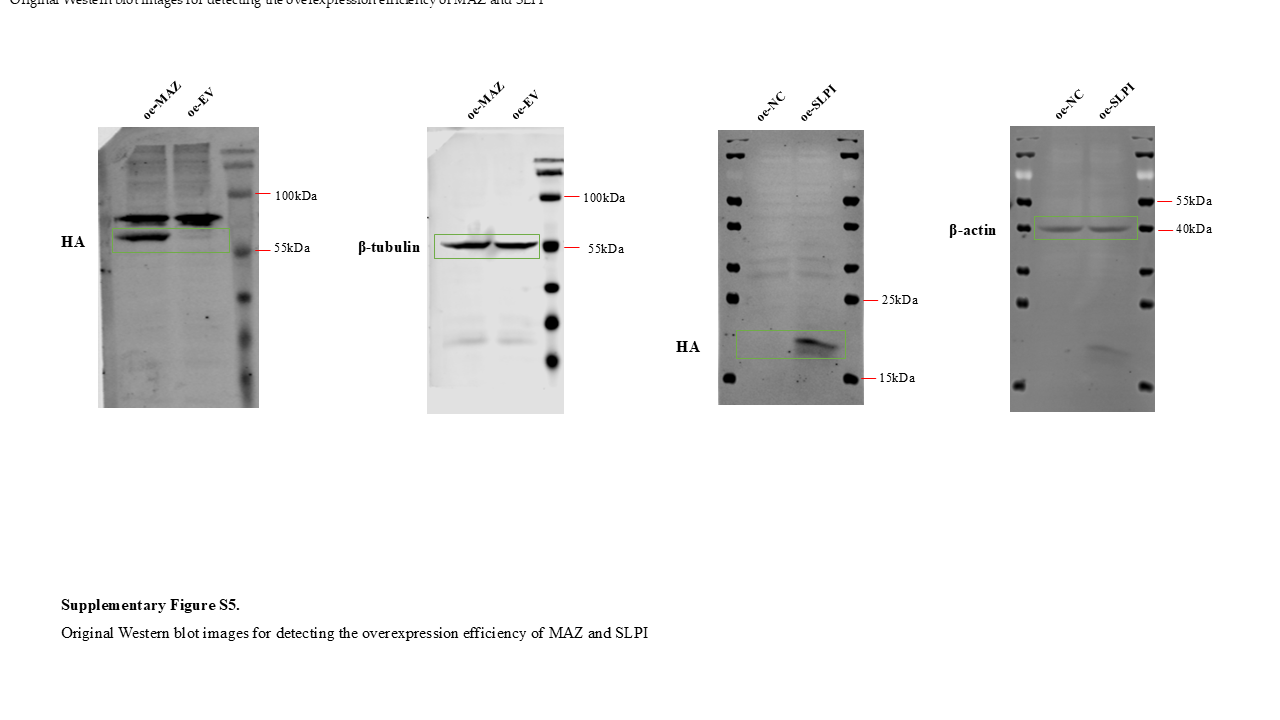

Supplement: Supplementary file 1 [file vetsci-13-00709-s001.zip › Supplementary Figure S5/Original Western blot images for detecting the overexpression efficiency of MAZ and SLPI.tif]

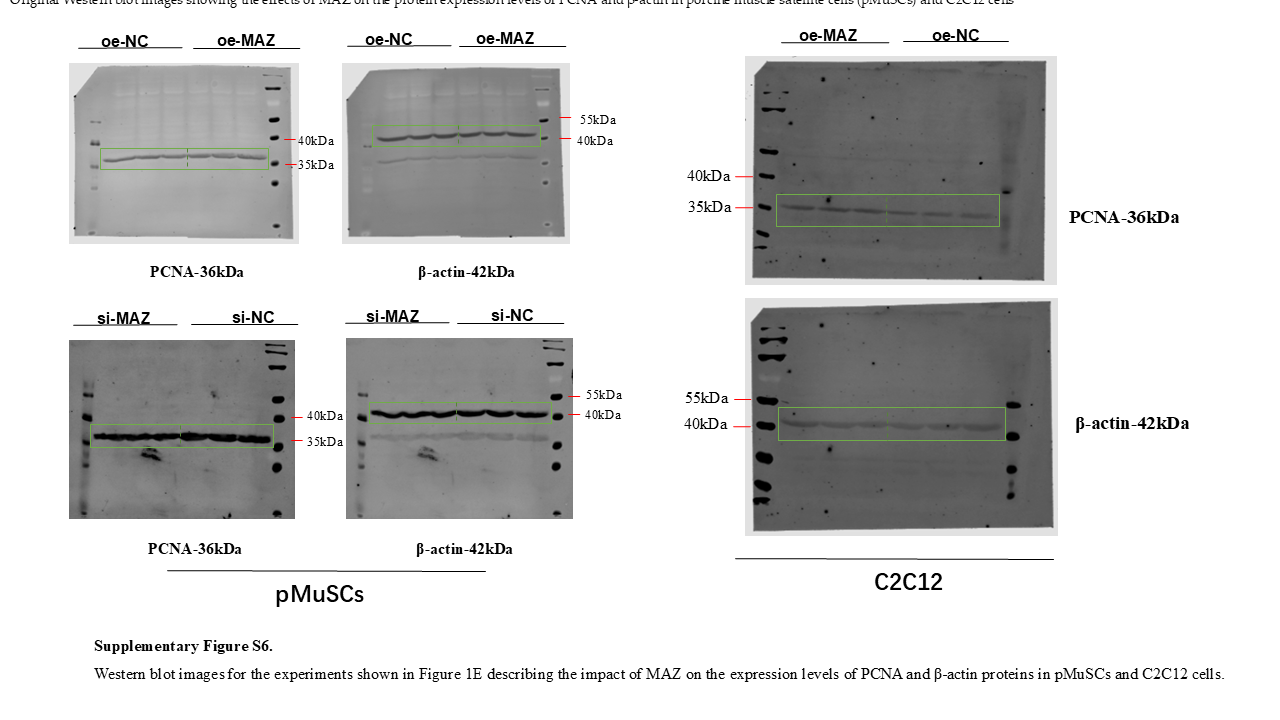

Supplement: Supplementary file 1 [file vetsci-13-00709-s001.zip › Supplementary Figure S6/Original Western blot images showing the effects of MAZ on the protein expression levels of PCNA and β-actin in porcine muscle satellite cells (pMuSCs) and C2C12 cells.tif]

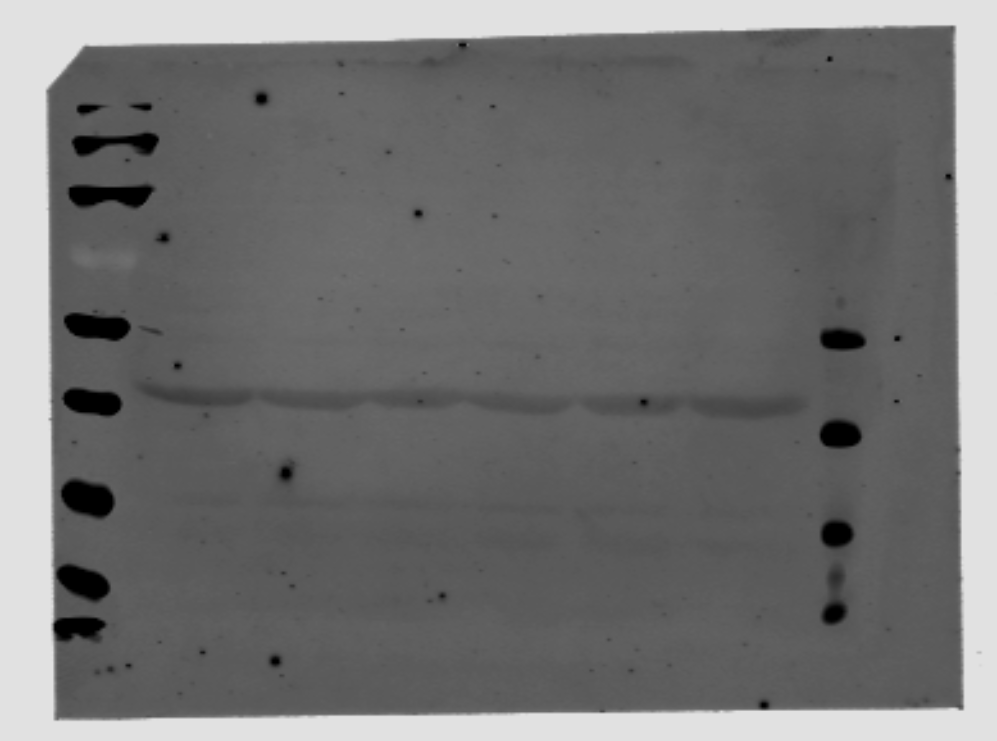

Supplement: Supplementary file 1 [file vetsci-13-00709-s001.zip › Supplementary Figure S6/The effect of MAZ overexpression and interference on PCNA/oe-MAZ-actin-C2C12-3.tiff]

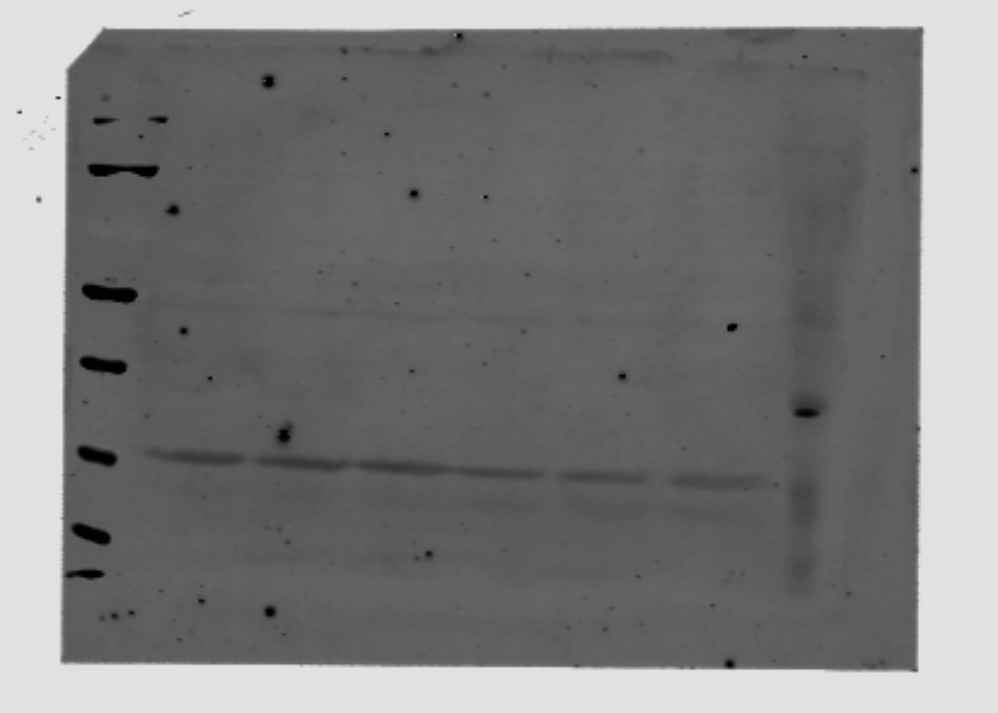

Supplement: Supplementary file 1 [file vetsci-13-00709-s001.zip › Supplementary Figure S6/The effect of MAZ overexpression and interference on PCNA/oe-MAZ-PCNA-C2C12-3.tiff]

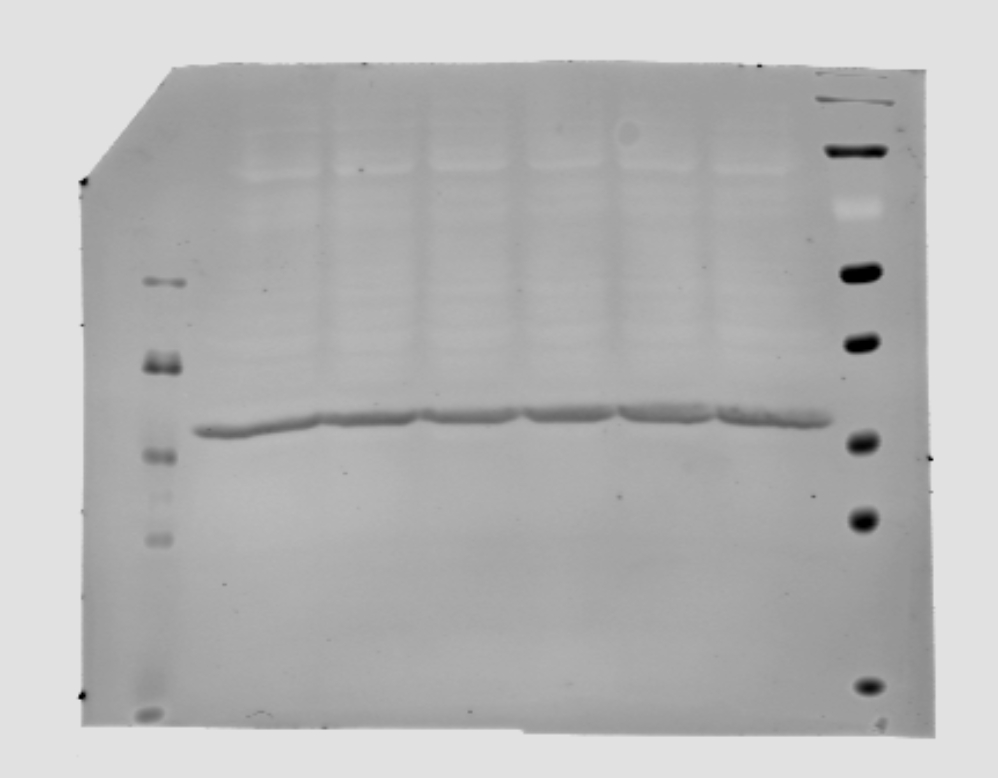

Supplement: Supplementary file 1 [file vetsci-13-00709-s001.zip › Supplementary Figure S6/The effect of MAZ overexpression and interference on PCNA/oe-MAZ-PCNA-pMuSCs-1.tiff]

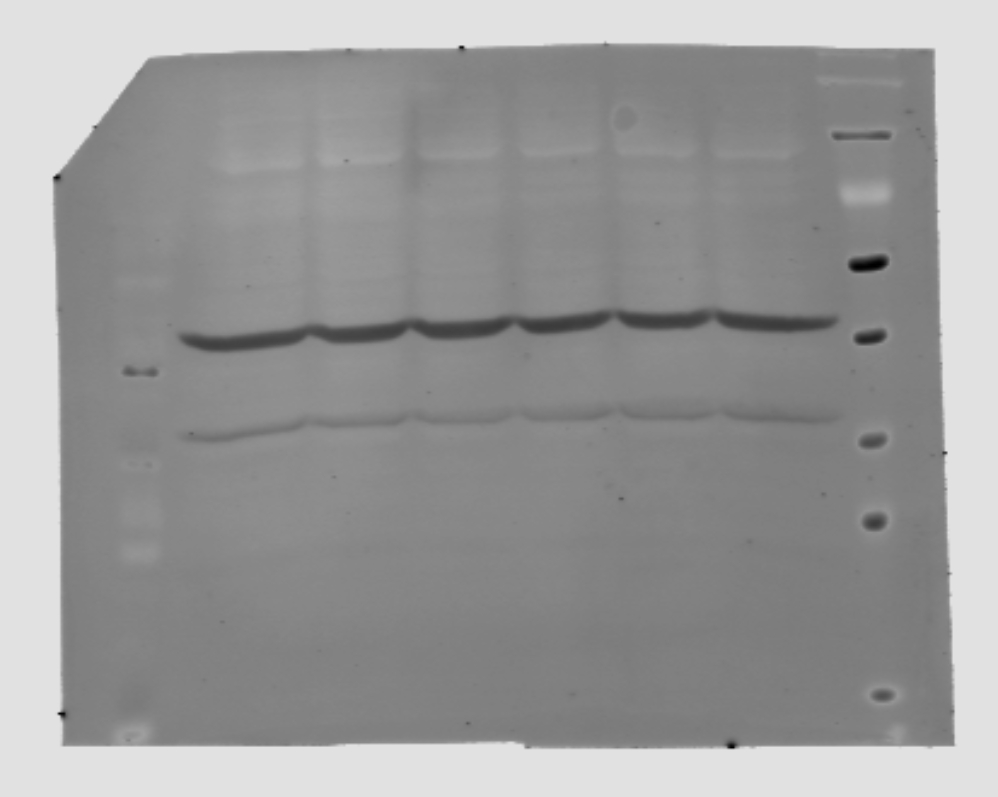

Supplement: Supplementary file 1 [file vetsci-13-00709-s001.zip › Supplementary Figure S6/The effect of MAZ overexpression and interference on PCNA/oe-MAZ-β-actin-pMuSCs-1.tiff]

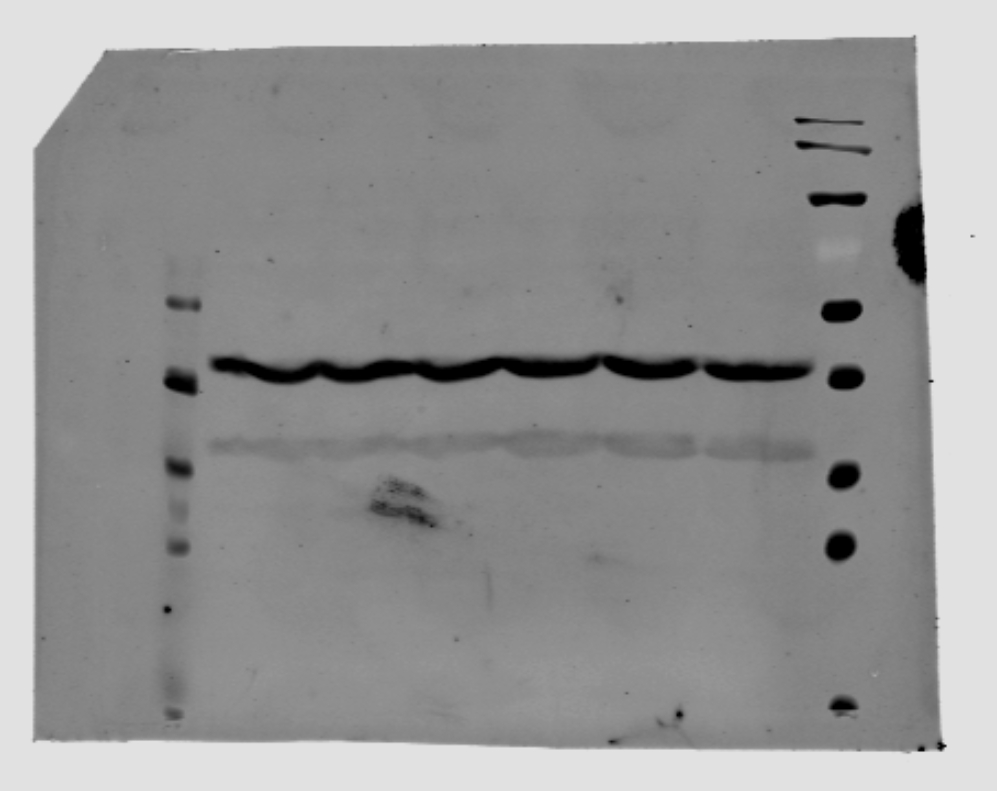

Supplement: Supplementary file 1 [file vetsci-13-00709-s001.zip › Supplementary Figure S6/The effect of MAZ overexpression and interference on PCNA/si-MAZ-actin-pMuSCs-2.tiff]

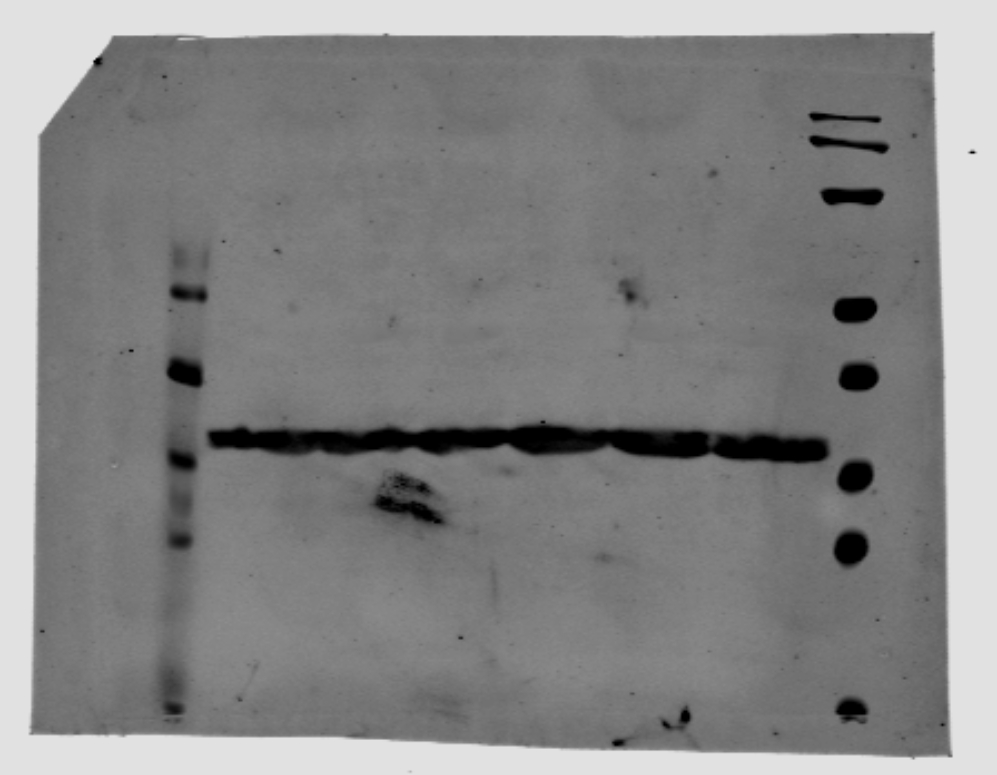

Supplement: Supplementary file 1 [file vetsci-13-00709-s001.zip › Supplementary Figure S6/The effect of MAZ overexpression and interference on PCNA/si-MAZ-PCNA-pMuSCs-2.tiff]

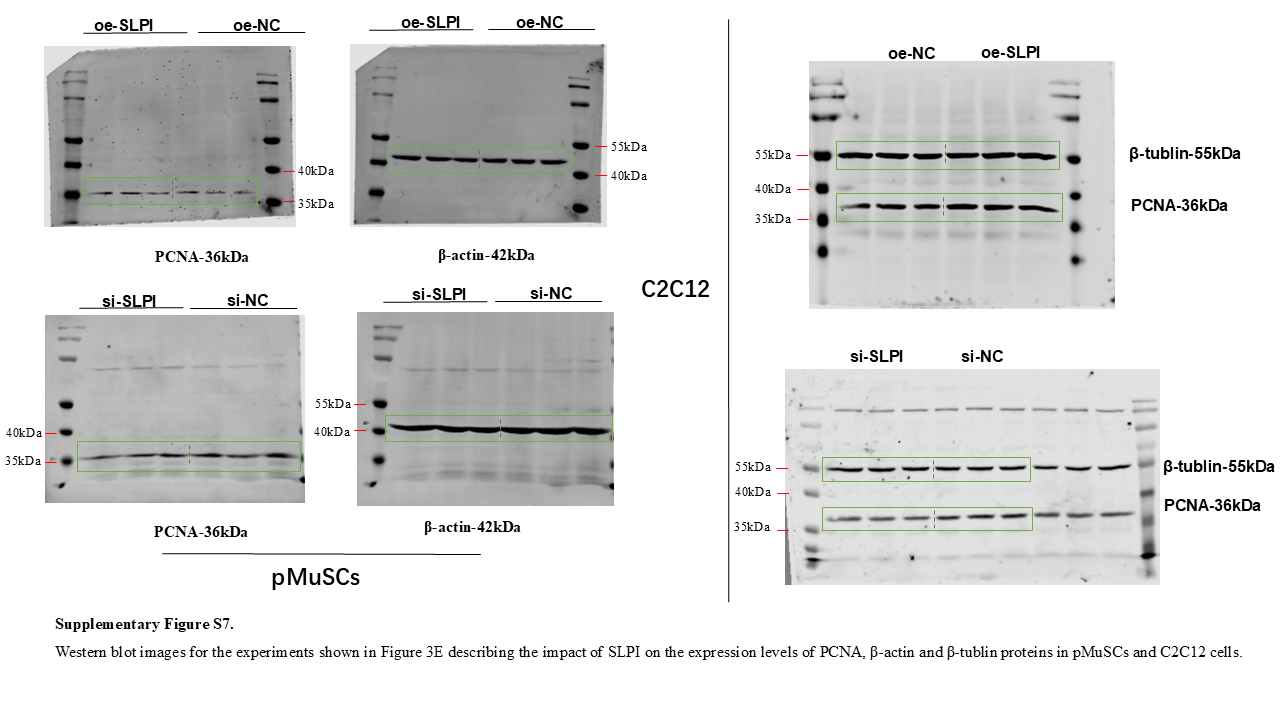

Supplement: Supplementary file 1 [file vetsci-13-00709-s001.zip › Supplementary Figure S7/Original Western blot images showing the effects of SLPI on the expression levels of PCNA, β-actin and β-tubulin in porcine muscle satellite cells (pMuSCs) and C2C12 cells.tif]

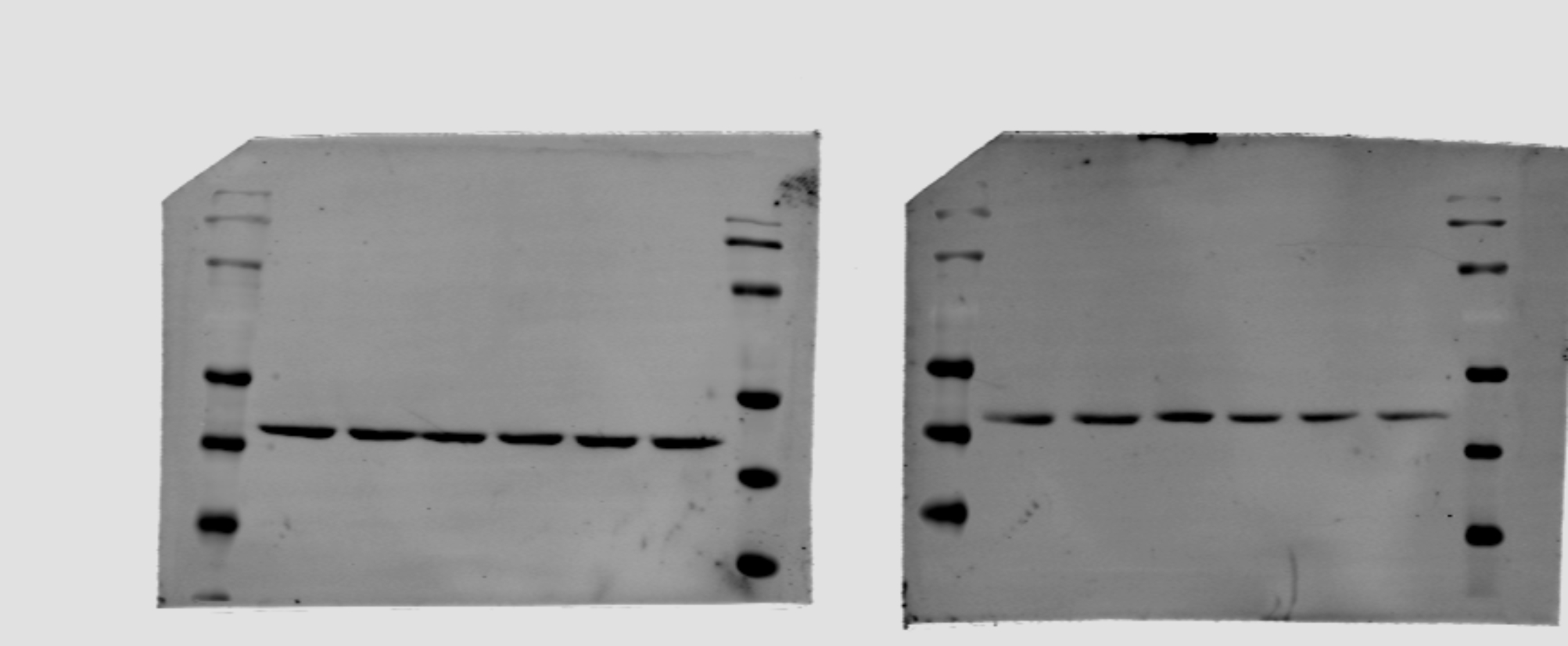

Supplement: Supplementary file 1 [file vetsci-13-00709-s001.zip › Supplementary Figure S7/The effect of SLPI overexpression and interference on PCNA/oe-SLPI-actin-pMuSCs-1.tiff]

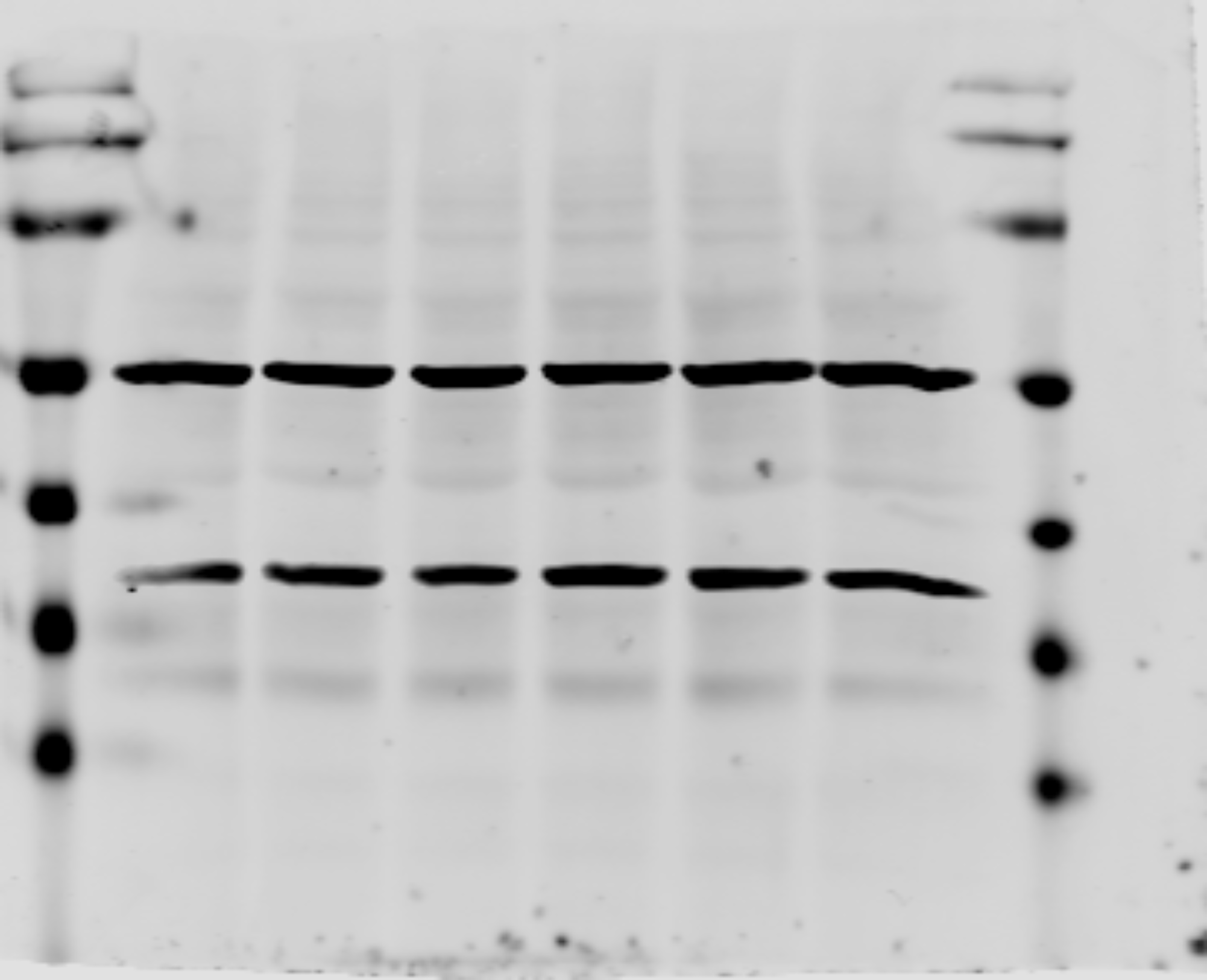

Supplement: Supplementary file 1 [file vetsci-13-00709-s001.zip › Supplementary Figure S7/The effect of SLPI overexpression and interference on PCNA/oe-SLPI-PCNA+tublin-C2C12-3.tiff]

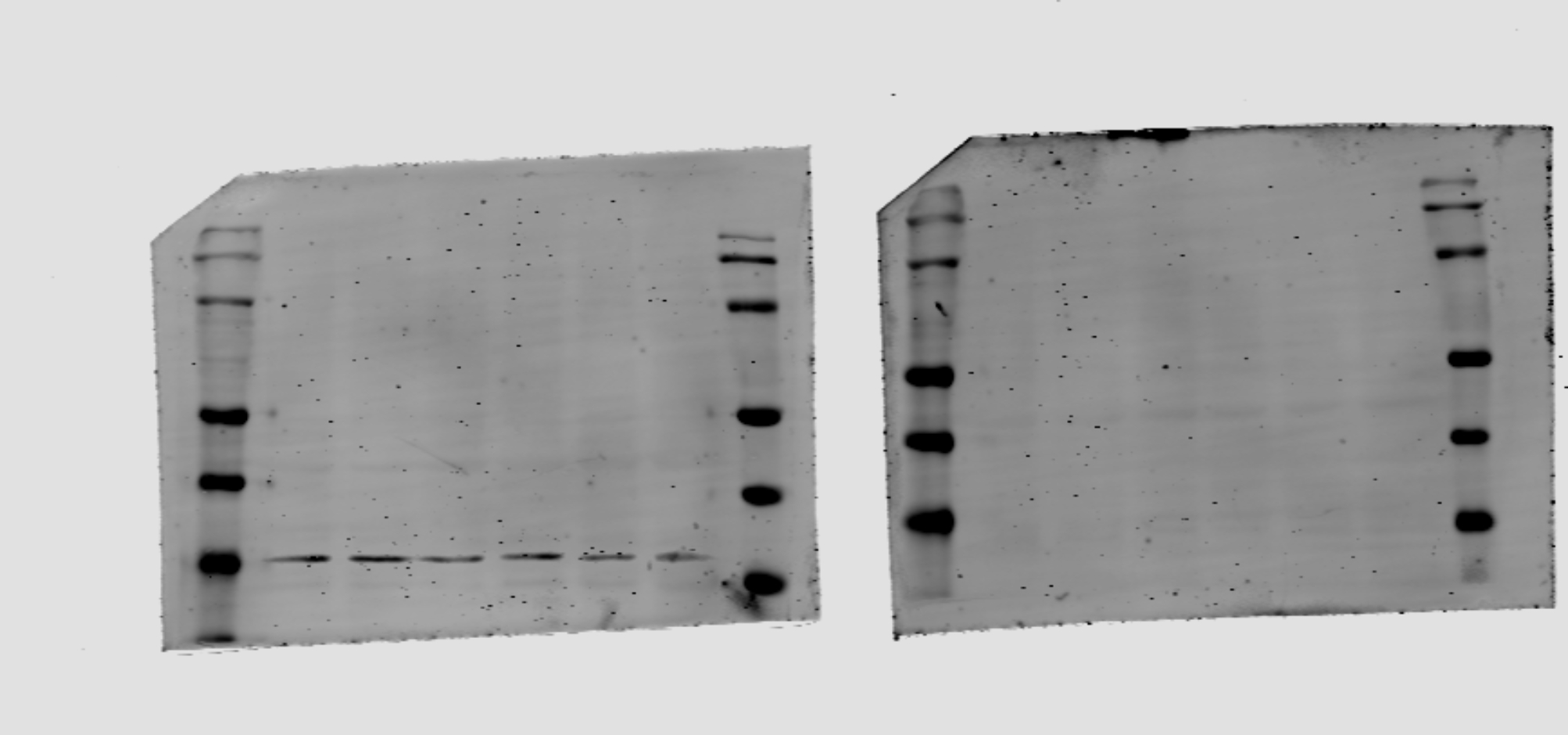

Supplement: Supplementary file 1 [file vetsci-13-00709-s001.zip › Supplementary Figure S7/The effect of SLPI overexpression and interference on PCNA/oe-SLPI-PCNA-pMuSCs-1.tiff]

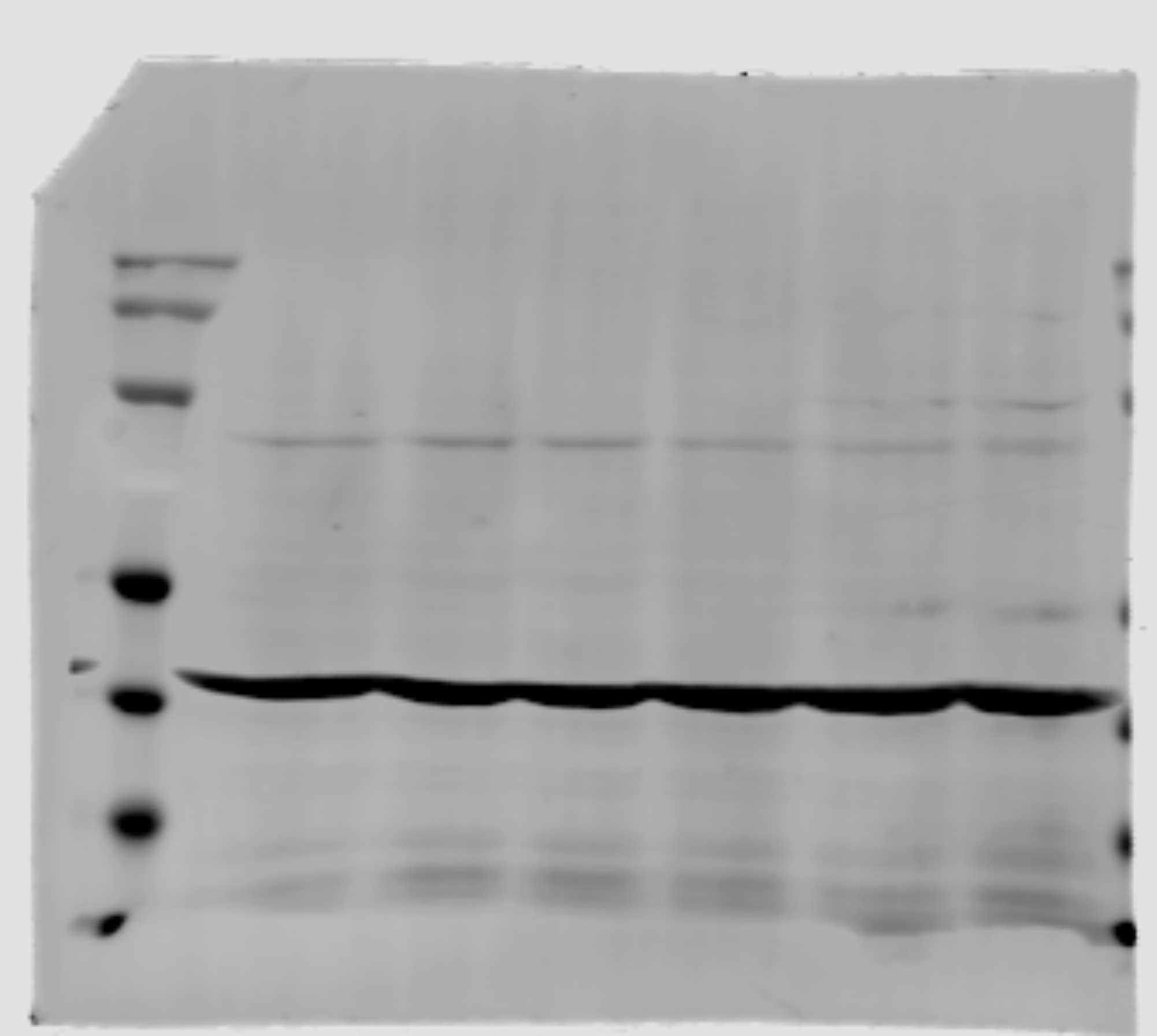

Supplement: Supplementary file 1 [file vetsci-13-00709-s001.zip › Supplementary Figure S7/The effect of SLPI overexpression and interference on PCNA/si-SLPI-actin-pMuSCs-2.tiff]

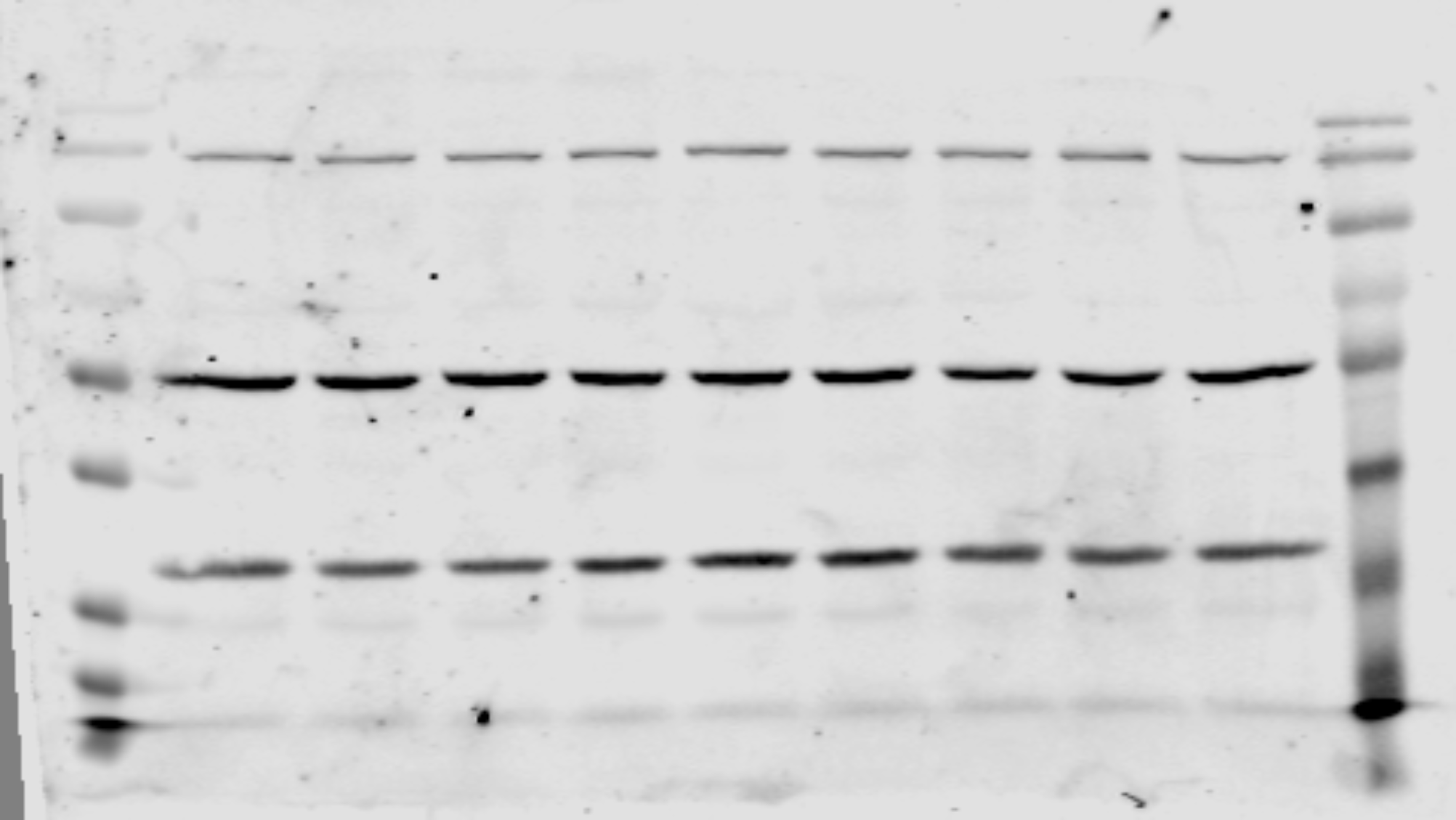

Supplement: Supplementary file 1 [file vetsci-13-00709-s001.zip › Supplementary Figure S7/The effect of SLPI overexpression and interference on PCNA/si-SLPI-PCNA+tublin-C2C12-4.tiff]

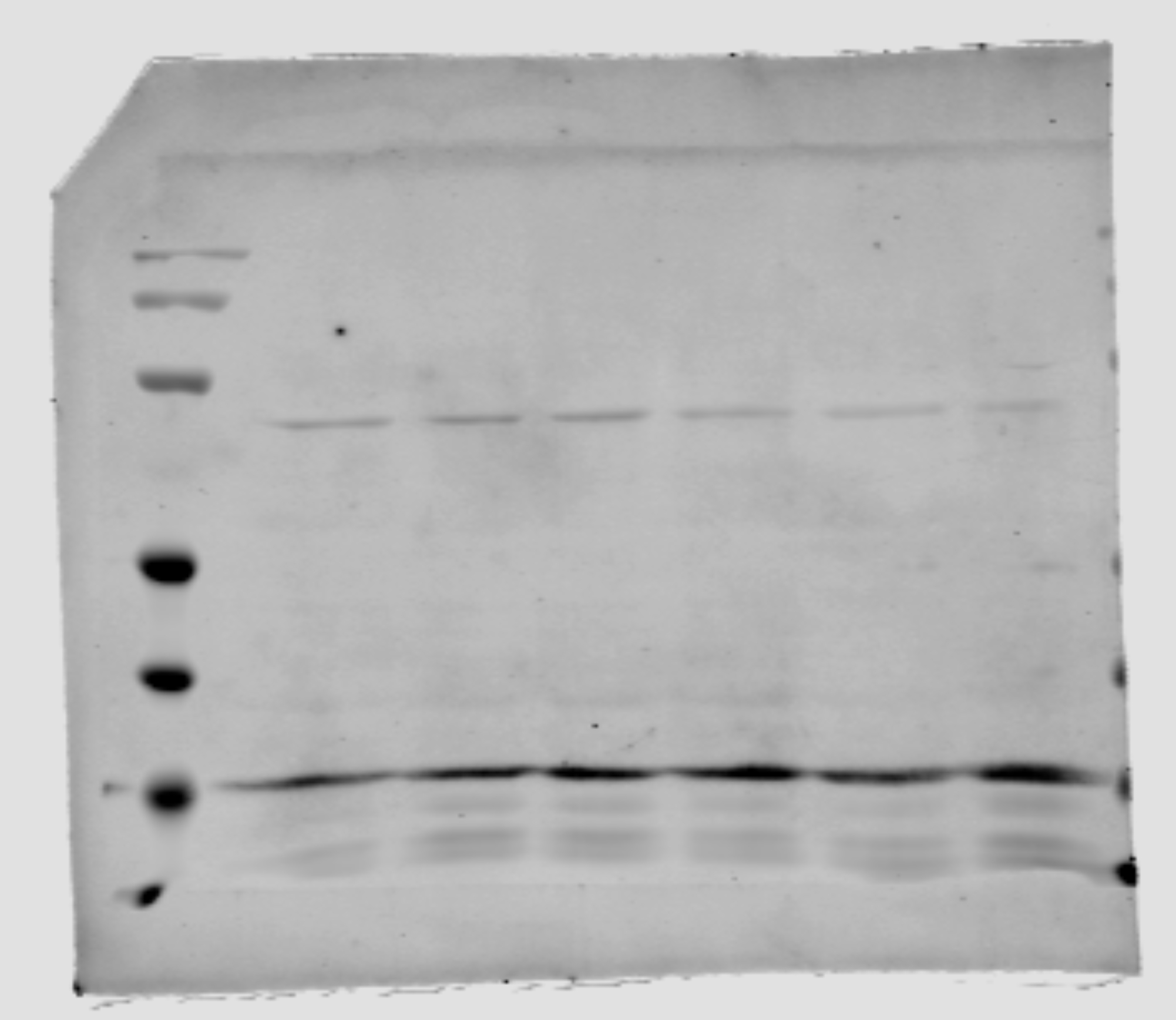

Supplement: Supplementary file 1 [file vetsci-13-00709-s001.zip › Supplementary Figure S7/The effect of SLPI overexpression and interference on PCNA/si-SLPI-PCNA-pMuSCs-2.tiff]
